# Beyond canonical PROTAC: biological targeted protein degradation (bioTPD)

**DOI:** 10.1186/s40824-023-00385-8

**Published:** 2023-07-21

**Authors:** Huifang Wang, Runhua Zhou, Fushan Xu, Kongjun Yang, Liuhai Zheng, Pan Zhao, Guangwei Shi, Lingyun Dai, Chengchao Xu, Le Yu, Zhijie Li, Jianhong Wang, Jigang Wang

**Affiliations:** 1 Shenzhen Institute of Respiratory Disease, Shenzhen Clinical Research Centre for Respirology, The Second Clinical Medical College, The First Affiliated Hospital, Shenzhen People’s Hospital, Jinan University, Southern University of Science and Technology, Shenzhen, 518020 Guangdong P. R. China; 2 School of Pharmaceutical Science, Southern Medical University, Guangzhou, 510515 P. R. China; 3 The Second Clinical Medical College, The First Affiliated Hospital, Shenzhen People’s Hospital, Jinan University, Southern University of Science and Technology, Shenzhen, 518020 Guangdong P. R. China; 4 State Key Laboratory for Quality Ensurance and Sustainable Use of Dao-di Herbs, Artemisinin Research Center, and Institute of Chinese Materia Medica, China Academy of Chinese Medical Sciences, Beijing, 100700 P. R. China; 5 Shenzhen Mental Health Center, Shenzhen Kangning Hospital, Shenzhen, 518020 Guangdong P. R. China

**Keywords:** Biological targeted protein degradation (bioTPD), Peptide, Antibody, Fusion protein, Nucleic acid

## Abstract

Targeted protein degradation (TPD) is an emerging therapeutic strategy with the potential to modulate disease-associated proteins that have previously been considered undruggable, by employing the host destruction machinery. The exploration and discovery of cellular degradation pathways, including but not limited to proteasomes and lysosome pathways as well as their degraders, is an area of active research. Since the concept of proteolysis-targeting chimeras (PROTACs) was introduced in 2001, the paradigm of TPD has been greatly expanded and moved from academia to industry for clinical translation, with small-molecule TPD being particularly represented. As an indispensable part of TPD, biological TPD (bioTPD) technologies including peptide-, fusion protein-, antibody-, nucleic acid-based bioTPD and others have also emerged and undergone significant advancement in recent years, demonstrating unique and promising activities beyond those of conventional small-molecule TPD. In this review, we provide an overview of recent advances in bioTPD technologies, summarize their compositional features and potential applications, and briefly discuss their drawbacks. Moreover, we present some strategies to improve the delivery efficacy of bioTPD, addressing their challenges in further clinical development.

## Targeted degradation pathways

Proteostasis is a highly complex and interconnected process that is closely linked to the normal growth and development of cells and tissues. It involves the correct folding, translocation, and elimination of proteins in eukaryotic cells [1]. The integrity of the cellular protein state is closely related to the activities of human life. Protein dysfunction, which includes misfolding and abnormal aggregation, is associated with a range of increasingly common human diseases, including Alzheimer’s disease (AD), Parkinson’s disease (PD), type II diabetes, systemic amyloidosis, and various tumors [2, 3].

To manage various abnormal proteins, eukaryotic cells evolve an elaborate system of protein regulation, which includes lysosomes, ubiquitin proteasomes, various chaperones, etc. [4, 5]. The system constantly monitors intracellular protein changes and processes abnormal proteins in time to avoid their pathological folding and aggregation. The proteasome system and lysosomal pathway represent the two most significant degradation pathways in cells. In particular, ubiquitin proteasomes degrade short-lived and soluble misfolded proteins [5], while lysosomes degrade long-lived proteins, insoluble protein aggregates, and intracellular parasites [6, 7]. In addition to the above degradation pathways, the ribonuclease (RNase) pathway and ClpCP proteases pathway are also of great importance in mediating proteostasis. The RNase-mediated modulators act upstream, targeting RNAs that encode disease-related proteins, and eventually influence protein abundance at the endpoint. As for the ClpCP proteases system, it is proteolytic machinery in some bacteria serving as the functional equivalent of the eukaryotic proteasomes [8].

In principle, these degradation pathways, except the RNase pathway for RNA degradation, make up the basis for targeted protein degradation (TPD). By harnessing the cell’s disposal system, TPD represents a promising therapeutic modality that allows access to most proteins of choice, requiring only a target binder (also called protein degrader) to carry out its intended role. While conventional pharmacological agents such as small molecule inhibitors and antibodies modulate fewer than 20% of the proteome, TPD offers a distinct means to address the rest of the unexplored, undruggable proteome with high selectivity. Through this, a disease can be alleviated or cured by reducing the number of harmful proteins rather than trying to modify or inhibit their functions. Moreover, Some kinds of cancer drug resistance mechanisms, such as gene mutation or overexpression could be conquered by TPD [9].

The concept of TPD was first proposed in 1999 [10]. Crews and his coworkers gave a more specific proof-of-concept of TPD in 2001 and founded Arvinas in 2013, the first company focusing solely on TPD. As TPD technologies have advanced rapidly over the past two decades, numerous types of degraders have showcased the efficacy, versatility, and transformative advantages of TPD. In this review, we will briefly introduce cellular degradation pathways and corresponding TPD technologies. The representative TPD technologies in terms of their chemical components, target ranges, advantages, and potential disadvantages are summarized in Table 1.

**Table 1:** Summary of representative TPD technologies related to different degradation pathways

Pathway	TPD technologies	Target range	Composition	Advantages	Potential problems	Year	Refs
**Proteasome**	PROTAC	Intracellular	Small molecule/ biomacromolecule/ hybrid structure	Relatively high selectivity; Acceptable oral bioavailability; Clear degradation mechanism; Catalytic and sub-stoichiometric	Poor solubility for small-molecule PROTAC; Poor cell permeability; Poor PK properties; Limited target spectrum	2001	[11, 12]
	Molecular glue	Intracellular	Small molecule	Acceptable oral bioavailability.	Difficult to design	2010	[13]
	SNIPER	Intracellular	Small molecule	Simultaneous degradation of POIs and IAPs; High specificity	E3 ligase IAPs dependently	2010	[14]
	HyT	Intracellular/ extracellular	Small molecule/ Small-molecule peptide conjugate	Some hydrophobic tags are independent of E3 ligases and ubiquitination; Wide range of potential targets;	Incomplete POIs degradation; Unclear degradation mechanism; Potential off-target effects	2011	[15]
	Trim-away	Intracellular	Antibody	High specificity; Rapid degradation speed	Need extra Trim21; Unable to recycle	2017	[16]
**Endosome-** **lysosome**	LYTAC	Extracellular/ membrane proteins	Antibody	Degrade extracellular and membrane proteins; High controllability	Limited shuttle receptors; Potential immunogenicity; Non-catalytic; Low degradation efficiency	2020	[17, 18]
	AbTAC	Membrane proteins	Bispecific antibody	Degrade membrane proteins; High specificity	Large molecular weight	2021	[19]
	GlueTAC	Extracellular/ membrane proteins	Nanobody-peptide conjugate	High specificity; Sufficient membrane permeability by a cell penetration peptide	Short half-life in vivo	2021	[20]
	Bispecific Aptamer Chimeras	Membrane proteins	Aptamer	Easy to design and prepare; Good stability	Low delivery efficacy; Short half-life in vivo	2021	[21]
	Sweeping antibody	Extracellular	Antibody	Allow recycling;	Required engineering for each target	2013	[22]
	Seldegs	IgG	Antigen-Fc fusion proteins	Degrade autoantibodies; Lower dose	Required engineering for each target; Antigen selection	2017	[23]
**Autophagy-lysosome**	CMA-based degrader	Intracellular/ membrane proteins/aggregates	Chimeric polypeptides.	High specificity; High degradation efficacy	Low delivery efficacy; Low stability; Limited therapeutic effects;	2014	[24]
	AUTAC	Intracellular/ damaged organelles	Small molecule-poly(A) oligonucleotide conjugate	A wide range of potential targets; Proteasome-independent	Low degradation speed; Potential off-target effects; Dependent on K63 ubiquitination;	2019	[25]
	ATTAC	Intracellular/ non-protein	Small molecule	A wide range of potential targets; Blood-brain barrier permeability;	Difficult to design	2019	[26, 27]
	AUTOTAC	Intracellular/ protein aggregates	Small molecule	Degrade protein aggregates	Low degradation speed	2022	[28]
**Ribonuclease**	RIBOTAC	RNA	Small molecule/small molecule-poly(A) oligonucleotide conjugate	Expand targeted range to RNA; High degradation efficacy	Difficulties in finding specific ligands for targeting RNA	2018	[29, 30]
**ClpCP proteases**	BacPROTAC	Bacterial proteins	Small molecule/small molecule-peptide conjugate	Expand the targeted range to bacterial protein	Low efficiency	2022	[8]

Abbreviations: POI, Protein of interest; IAPs, Inhibitor of apoptosis protein; HyT, Hydrophobic Tag; PROTAC, Proteolysis Targeting Chimeras; TPD, Targeted protein degradation; PK, Pharmacokinetics; SNIPER, Specific and Non-genetic IAP-dependent Protein Erasers; Trim21, Tripartite motif-containing protein 21; LYTAC, Lysosome-targeting chimeras; AbTAC, Antibody-based PROTAC; IgG, immunoglobulin G; CMA, Chaperone mediated autophagy; AUTAC, Autophagy-targeting chimera; ATTAC, Autophagy-tethering compounds; AUTOTAC, AUTOphagy-TArgeting Chimera; RIBOTAC, Ribonuclease targeting chimera

### Ubiquitin-proteasome pathway

Aberrations in the ubiquitin system have been implicated in the pathogenesis of neurodegenerative diseases, Huntington’s disease, type II diabetes, and cancers [31–33]. As a key regulator of eukaryotic protein homeostasis, the ubiquitin-proteasome system degrades disease-associated misfolded, abnormally aggregated proteins. Clearly, this system has the potential to be applied for the removal of disordered proteins as a strategy for drug development.

Ubiquitin (Ub) is a highly conserved 76-amino acid protein, which links to substrate proteins via their lysine residues (mainly K63 and K48) [10] as a modifier, in a process called ubiquitination. Ubiquitination is a significant posttranslational protein modification in eukaryotic cells. In addition to engaging protein degradation, ubiquitination also plays a major role in regulating a broad host of cellular processes, including protein transport, cell cycle, DNA repair, apoptosis, and signal transduction [34, 35]. The ubiquitin-proteasome system comprises E1 (ubiquitin-activating enzyme), E2 (ubiquitin-conjugation enzyme), and E3 (ubiquitin ligase) enzymes, as well as the 26 S proteasome [36, 37]. Ub is attached to the lysine residues of substrate proteins through a sequential process involving these three enzymes. First, a high-energy thioester bond attaches Ub to the E1 in an ATP-consuming manner. Then, the activated Ub is transferred to the active site cysteine of E2. E3, the third enzyme, works with E2 to catalyze the transfer of (poly)ubiquitin to the protein that is marked for degradation [38, 39]. Ultimately, the 26 S proteasome receives the polyubiquitinated protein and degrades them into small peptides.

**Fig. 1:**
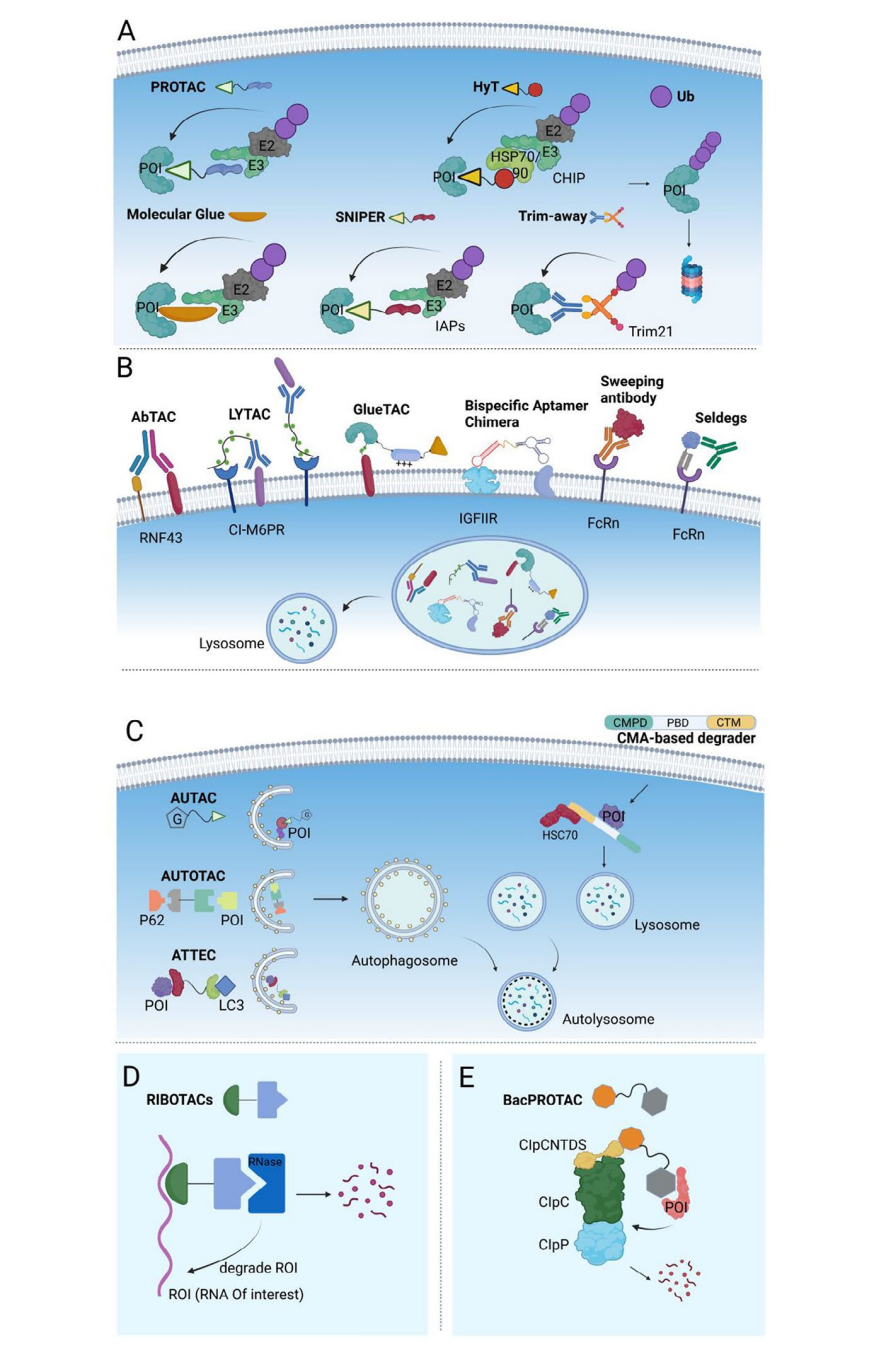
Targeted degradation via five distinct degradation pathways. (**A**) Proteasome pathway. Molecule glue is a monovalent small molecule degrader that employs a single interaction with the POI or an E3 ligase, whereas PROTAC, hydrophobic tags (HyT), and SNIPER are chimeric molecules that simultaneously bind to the POI and the E3 ubiquitin ligase. These degraders enable POI ubiquitination and subsequent proteasomal degradation. TRIM-away system consists of an antibody and TRIM21. TRIM21, an E3 ligase recognizing the Fc fragment of an antibody, can facilitate the antibody-POI complex or antibody-bound pathogens to the proteasome for degradation. (**B**) Endosome-lysosome pathway. AbTAC, LYTAC, bispecific aptamer chimeras, GlueTAC, sweeping antibody, and Seldegs develop an ‘outside-in’ strategy to shutter extracellular/membrane POIs to the endosome and undergo lysosomal degradation. (**C**) Autophagy-lysosome pathway. AUTAC, ATTEC, and AUTOTAC, also chimeric molecules, link the intracellular substrate and adaptor proteins (e.g. LC3, p62) or autophagosome, which was fused with lysosome and processed to degradation. CMA-based degraders degrade membrane/intracellular proteins by harnessing chaperone-mediated autophagy (CMA), rather than macroautophagy. (**D**) Ribonuclease pathway. RIBOTAC is a targeted RNA degradation technology, which recruits a nuclease to a specific RNA and triggers its collapse. (**E**) ClpCP proteases pathway (bacterial degradation machinery). BacPROTAC tethers the target bacterial protein to the ClpC:ClpP protease and then primes the neo-substrates for degradation. The figure was created in BioRender.com

Due to their capacity to facilitate the ubiquitination of substrate proteins and drive proteasomal degradation, E3 becomes a primary focus of research in TPD. In 2001, the group of Crews [11] created the first proteolysis-targeting chimera (PROTAC) using the Skp1-Cullin-F-box (SCF) that targets methionine aminopeptidase-2 (METAP2) for degradation. By designing E3 ligands and linking them to target protein conjugates, multiple ubiquitin proteasome-based TPD strategies have been created to degrade specific proteins [40–42]. Among them, PROTAC, hydrophobic tags (HyT) [15], and specific and non-genetic inhibitors of apoptosis protein-dependent protein erosive agents (SNIPER) [14] are bispecific chimeric molecules that simultaneously bind to the proteins of interest (POIs) and E3, enabling POIs ubiquitination and subsequent proteasomal degradation. Distinct from the above degraders, molecular glues are small chemicals that bind to only the ligase (in most cases) or the POI and induce proteasomal degradation [13, 43]. The TRIM-away system utilizes tripartite motif-containing protein 21 (TRIM21, an E3 ligase recognizing the Fc fragment of an antibody) to target the antibody-POI complex or antibody-bound pathogens to the proteasome for degradation [16] (Fig. 1A).

### Lysosomal degradation pathway

Proteasome-mediated TPD has become a powerful tool in modulating undruggable protein targets. However, the scope of the present proteasome-dependent TPD technologies is virtually limited to soluble intracellular proteins [16]. Similar to the ubiquitin-proteasome system, the lysosomal system is also crucial for maintaining protein homeostasis and the integrity of the intra- and extra-cellular environment [44]. Compared to the proteasomal system, a wider range of substrates, including soluble proteins, aggregated proteins, non-proteinous components, and even organelles, can be degraded by lysosomes. Encouragingly, lysosomal degradation has become an emerging modality for TPD technology and an alternative strategy for degradation techniques based on the ubiquitin-proteasome system.

In recent years, TPD methods that use the lysosomal degradation pathway, such as antibody-based PROTAC (AbTAC), lysosome-targeting chimeras (LYTAC), GlueTAC, bispecific aptamer chimeras, AUtophagy-targeting chimeras (AUTAC) or, AUTOphagy-targeting chimeras (AUTOTAC), and autophagy-tethering compounds (ATTEC) have emerged [10, 45]. Endosome-lysosome and autophagy-lysosome are two lysosomal degradation pathways commonly involved in TPD.

Endocytosis is a general process by which the plasma membrane folds and engulfs external materials into a vesicle. The filled vesicle then undergoes a series of procedures to become an endosome and eventually fuses with lysosomes to digest vesicular cargo [46]. The endocytic uptake of the fluid-phase is associated with clathrin-mediated endocytosis, caveolin-mediated endocytosis, clathrin/caveolae-independent endocytosis, and macropinocytosis, etc. [47]. Phagocytosis is a special form of endocytosis, in which cells transport harmful substances, such as bacteria, viruses, and various pathogens, by phagocytosis to the lysosomes for degradation, which protects the cells from harmful external substances [48].

With intensive ongoing research on the endocytic lysosomal pathway, the TPD scope has been greatly expanded from intracellular targets to extracellular and membrane targets through a series of new TPD strategies harnessing this pathway, such as AbTAC (a bispecific antibody targeting a transmembrane E3 ligase and a membrane-related protein) [19], LYTAC (an antibody/small molecule targeting a POI and a lysosomal shuttle receptor) [17, 18], GlueTAC (a covalent nanobody fused to a cell-penetrating peptide/lysosome sorting sequence (CPP-LSS)) [20], bispecific aptamer chimeras (a bispecific aptamer chimera binding to a lysosomal shuttle receptor and a transmembrane protein) [21], sweeping antibody [22] and Seldeg [23] (both engineered antibodies hijacking the Fc receptors) (Fig. 1B).

Apart from the endosome-lysosome pathway, the autophagy-lysosome pathway furnishes another avenue in TPD. To maintain intracellular homeostasis and normal metabolic activities, autophagy, a highly conserved degradation mechanism in yeast and mammals, breaks down dysfunctional intracellular proteins and damaged organelles to generate nutrients, such as amino acids and lipids, that can be recycled by cells [49, 50]. Macroautophagy, microautophagy, and chaperone-mediated autophagy (CMA) are three specific forms of the autophagic lysosomal pathway [51].

Broadly referred to as autophagy, macroautophagy begins with a detached membrane structure called the phagophore, which is derived from a phospholipid bilayer containing lipidated LC3. This phagophore enlarges to engulf autophagic substrates, including proteins and organelles, sequestering them in a double-membrane vesicle known as the autophagosome. Cargo degradation occurs after the laden autophagosomes are fused with lysosomes [52]. Microautophagy is a non-selective phagocytic process in which the lysosomal membrane directly engulfs cytoplasmic cargos and during that process, they are degraded by multiple hydrolases [53]. During CMA, proteins with specific motifs (KFERQ) are selected by their chaperones, targeted to lysosomes, and directly translocated into the lysosomal lumen for cargo clearance [54].

AUTAC, which compromises an S-guanine tag and a warhead for intracellular POI via a flexible linker, was the first degrader to be developed targeting the autophagy machinery. AUTAC recruits autophagosomes through K63 polyubiquitination and destines substrates for selective autophagy [25] (Fig. 1C). Differing from AUTAC which uses selective autophagy (xenophagy) for degradation, ATTEC and AUTOTAC directly engage the autophagy pathway. LC3 (Atg8) and p62, two widely used markers of autophagy, are closely related to autophagy initiation and have been used to develop ATTEC and AUTOTAC, in which an ATTEC molecule simultaneously binds to LC3 and a POI [26], while an AUTOTAC molecule binds to p62 and a POI [28]. Subsequently, these chimeric architectures recruit autophagosomes and lead to consecutive autophagy-mediated degradation (Fig. 1C). The CMA-based degrader [24], typically a peptide-based degrader, contains a cell membrane penetrating domain (CMPD), a protein binding domain (PBD), and a CMA sorting signal. This fused peptide drives protein clearance through the CMA pathway (Fig. 1C).

### Alternative targeted degradation pathways

#### RNase pathway

The Encode program has revealed that while only 1–2% of the human genome encodes proteins, at least 76% is transcribed into RNA [55]. As expected, non-coding RNA, including microRNA, lncRNA, and intron RNA, play pivotal roles in the regulation of gene and protein expression. Acting as crucial regulators of biological functions, RNA generation and elimination are tightly controlled. RNase is a class of nucleases that naturally regulate RNA lifetime. By exploiting RNases, it is promising to regulate RNA fates via chimeric structures similar to PROTACs.

Disney’s group has performed notable work in expanding the range of TPD from proteins to RNA. Using their previous Inforna method to design small molecules for targeting RNA [56], Disney’s group [29] developed the first RNase targeting chimera (RIBOTAC), in which a short 2’-5’ A_4_ oligonucleotide targeting RNase L was linked with a small molecule recruiting the primary transcript of microRNA-96 (pri-miR-96) (Fig. 1D). Importantly, the RIBOTAC degrader not only recruits inactive RNase L to the target RNA but also activates its catalytic activity upon their conjugation. In line with the demands of combating the COVID-19 pandemic, Disney’s group [30] designed a series of RIBOTACs to destroy the frameshifting element within SARS-CoV-2’s RNA genome in 2020. These RNA degraders are specifically bound to the revised attenuator hairpin structure of the viral RNA, suggesting a potential tool to target the SARS-CoV-2 RNA genome.

Compared with other RNA silencing technologies (antisense oligonucleotides and siRNA), RIBOTAC has several outstanding advantages, such as catalytic properties and better bioavailability [29].

#### ClpCP protease pathway

PROTACs eliminate specified proteins by engaging the eukaryotic ubiquitin-proteasome machinery. However, this promising technology has been restricted to the ubiquitination system in eukaryotes and cannot be applied in bacteria, which do not possess a ubiquitination system. Although ubiquitin is unique to eukaryotic cells, some prokaryotic cells also have similar degradation markers. A short fragment of phosphorylated arginine residues (pArg) acts as a hydrolysis tag which can be recognized by the bacterial ClpC:ClpP (ClpCP) protease system, the functional equivalent of the eukaryotic proteasome machine in gram-positive bacteria and mycobacteria [57]. Compared with the eukaryotic proteasome that recognizes cascaded polyubiquitin signals, the ClpCP protease recognition mechanism is much simpler: a pArg tag is attached to the target protein and then recognized by the ClpCP protease as a degradation signal [57].

Recently, Morreale et al. [8] developed the first bacterial PROTACs (BacPROTACs) redirecting the ClpCP protease for degrading neo-substrates, which expands TPD application to bacteria and provides a novel platform for antibiotics discovery (Fig. 1E). A pArg group or pArg-like cyclic peptides were chosen as the ligands for targeting the ClpC protease. Their structural study indicates that the protease ligands of BacPROTACs not only serve as targeting moieties but also convert ClpC into active, higher-order oligomers with ClpP. As expected, the designed BacPROTACs showed a high affinity for the protease and demonstrated an efficient degradation activity in vivo.

## Development and disadvantages of small molecule PROTACs

Crews et al. published the initial report on PROTAC technology based on the SCF in 2001 [11]. In order to target a protein to the SCF complex, a ligand of METAP2 (ovalicin) was coupled to the ligand of βTRCP E3 ligase (I kappa Bα (IκBα) phosphopeptide) via a linker. The PROTAC then promoted METAP2 ubiquitination and destruction in a cell-free system; these results served as the first proof-of-concept that PROTAC degradation of a target protein could be effective in vitro.

Later in 2003, Crews et al. employed this approach once again to degrade estrogen receptor (ER) and androgen receptor (AR) [12]. After being microinjected into 293GFP-AR cells, a dihydrotestosterone-IκBα phosphopeptide PROTAC molecule induced significant GFP-AR degradation. This finding demonstrated for the first time that PROTAC is not conceptually limited to extracellular space and can trigger protein degradation in cells via the proteasomal pathway.

For the construction of PROTACs, the IκBα phosphopeptide is used as a binder of the β-TRCP E3 ligase. Similar to this, researchers discovered a specific class of short peptides that bind to von Hippel-Lindau (VHL), which is an E3 ubiquitin ligase that targets the degradation of the tumor-associated transcription factor hypoxia-inducible factor 1 (HIF1) [58, 59]. The Zhang lab [60] created the Fumagillo/estradiol-octapeptide (a ligand of HIF1) PROTACs which target METAP2 and ER, and ubiquitination of the target protein was observed after treating cells with these compounds. Similarly, Schneekloth et al. [61] developed PROTACs containing a short peptide (heptapeptide) as a VHL ligand tethered to AP21998 or DHT, which targets FKBP12 or AR, respectively.

Technically, these early PROTACs are now defined as ‘bioPROTACs’ as they are not fully small-molecule chemicals, but instead, incorporate peptide ligands for targeting E3 [62]. Previous studies have demonstrated that it can be a challenge for an unmodified PROTAC with peptide chains, which have high polarity and poor permeability, to enter cells [63]. Additionally, the degradation effectiveness is impacted by the low stability of the peptide, which makes it susceptible to degradation and less effective [64]. Therefore, researchers have attempted to identify small molecules with similar peptide ligand functions and used them to bind E3 ligases to improve the pharmacokinetic properties and stability of PROTAC.

In 2008, the Crews lab [65] created the first heterobifunctional fully small-molecule PROTAC using mouse double minute 2 homolog (MDM2), an E3 ligase targeting p53. The PROTAC molecule was composed of SARM, a small-molecule AR ligand [66], and nutlin, a small-molecule MDM2 ligand [67]. Significant degradation of AR was observed in cells treated with the SARM-nutlin PROTAC. Unexpectedly, this small molecule PROTAC was highly cell-permeable, which caused the construction of numerous small molecule PROTACs to follow.

Inhibitor of apoptosis protein 1 (cIAP1) is the second E3 ligase after MDM2 used in the construction of small molecule PROTAC. Sekine et al. [68] reported a small molecule named ME-BS that selectively downregulates cIAP1. ME-BS directly interacts with the baculovirus IAP repeat 3 (BIR3) domain of cIAP1, promoting its ubiquitination activity as well as self-degradation. Subsequently, a variety of small-molecule PROTACs that harness ME-BS have emerged, such as ATRA-MEBS [69] and 4-OHT- MEBS [70].

A small molecule VHL-recruiting PROTAC has also been developed by replacing the HIF1 peptide with high-affinity small-molecule ligands [71, 72]. Subsequently, various VHL-based small molecule PROTACs were developed to target and degrade a variety of different target proteins, including receptor-interacting serine/threonine kinase 2 (RIPK2) [73], BCR-ABL [74], TANK-binding kinase 1 (TBK1) [75], epidermal growth factor receptor (EGFR) [76], tripartite motif containing 24 (TRIM24) [77], and bromodomain-containing 4 (BRD4) [78–80].

In addition to the E3 ligases mentioned above, small molecule PROTACs based on E3 ubiquitin ligases such as SCF^β−TRCP^, cereblon, RING finger protein 4 (RNF4), RING finger protein 114 (RNF114), and Kelch-1ike ECH- associated protein l (KEAP1) have also been developed [81–85]. Since the first example of small-molecule PROTAC was reported, the technology in the past two decades has been expanded from academia to industry, where several pharmaceutical companies have built pipelines for PROTACs potentially translated into clinical applications. This rapid development of small molecule PROTAC is due to favorable cell permeability, good stability, high degradation efficiency, and a longer duration of action [86–89].

Nevertheless, it becomes clear that small-molecule PROTAC has a few disadvantages: the small-molecule PROTAC (1) is heavily dependent on the binding pocket of the target protein [11]; (2) depends on the proteasome system to eliminate target proteins [90]; (3) displays off-target effects and potential adverse reactions [91, 92]; and (4) generates a Hook effect where saturating doses of PROTAC cause excessive production of non-productive binary complexes over ternary complexes and influence the degradation efficiency [93].

## Advances in bioTPD

Biological TPD (bioTPD), which includes the previously mentioned peptide-based PROTAC and other non-small molecule-based TPD technologies constituted of nucleic acids, or proteins [10, 45, 94], exhibits numerous advantages over small-molecule TPD. These include: (1) antibodies and peptides can specifically bind undruggable proteins and are not affected by the target protein binding pocket, which is more conducive to the construction of bioTPD [95]; (2) protein- and peptide-based bioTPD are less demanding to design and synthesize and display superior safety and less toxicity [96]; (3) the ligands of protein/peptides can recognize the mutated target [97], thus reducing off-target effects; (4) LYTAC, GlueTAC, nucleic acid PROTAC, and other TPDs that depend on the lysosomal pathway can degrade membrane proteins and extracellular proteins [10]; and (5) The ligand affinity and specificity of peptide or antibody (antibody fragments) is usually higher than that of small-molecule compounds, which may contribute to higher efficiency and selectivity of bioTPD. Actually, there are few studies on the comparison of the degradation speed/efficiency between bioTPD and small-molecule TPD, thus further research is warranted to comprehensively explore this issue. The availability of bioTPD further extends the canonical PROTAC toolbox, providing new options for protein degradation besides small molecule targeted degradation agents and warrants further development.

In this review, the bioTPD category is divided into four subgroups: peptide-, fusion protein-, antibody (fragments)-, and nucleic acid-based bioTPD. The following section will focus on the development of each subgroup, summarizing their components, mechanisms, features, and potential scenarios for application. Some limitations of bioTPD will also be issued.

### Peptide-based bioTPD

Peptide-based bioTPD, especially peptide PROTACs, is the earliest form of TPD technology to be developed. Given their undesirable pharmacokinetic profiles, research in peptide-based degraders moved at a slower pace than small-molecule degraders. However, peptide degraders are still used as complementary means for small-molecule degraders. Peptide-based bioTPD degraders are divided into whole peptides and hybrids that contain both a peptide ligand and a small-molecule warhead. Before small-molecule ligands for VHL, cereblon, and Keap1 were discovered, peptide ligands have been widely used for E3 targeting. Here, we mainly describe peptide degraders based on different E3 ligases and lysosomal adaptor proteins (Table 2).

#### VHL-dependent peptide bioTPD

##### (1) MLAP(OH)YIPM

To create a small molecule protein hydrolysis inducer (SMPI) that targets the ER and METAP2, Zhang et al. [60] combined estradiol/fumagillo with MLAP(OH)YIPM, a short HIF-derived peptide [98]. In lung cancer A549 cells and breast cancer MCF-7 cells, treatments with the above chimeras resulted in a considerable rise in METAP2 ubiquitination levels and ER degradation in a time-dependent manner. Rescue experiments demonstrated that the SMPI lost its protein degradation capability when Pro^OH^ was replaced by Ala. These results indicate that hydroxylated proline is necessary for its binding to VHL [85] and that this moiety is important for VHL-binding peptides.

Drawing on earlier research, Zhang et al. [60] changed the sequence MLAP(OH)YIPM to construct a pentapeptide structure by deleting the flanking amino acids while retaining Pro^OH^. The PROTAC (E2-penta), which was created by combining this pentapeptide with estradiol, also successfully increased ER degradation. These findings indicate that altering the peptide chain while keeping the vital Pro^OH^ group not only ‘simplifies’ PROTAC for more effective manufacturing, but also offers a theoretical foundation for the creation of more peptide mimics.

##### (2) LAP(OH)YI

In 2004, Zhang et al. [60] detailed that brief pentapeptide structures containing Pro^OH^ have VHL binding capacity. Inspired by this, Bargagna-Mohan et al. [99] examined the potential of Pro^OH^-based-domain-estradiol PROTACs containing peptide chains of diverse lengths (~ 5–8 amino acids), to debase ER at the tissue level by employing a three-dimensional (3D) endothelial cell germination assay (3D-ECSA). They found that PROTAC built using LAP(OH)YI exhibited a more efficient ER debasement capacity than PROTAC built using an octapeptide.

LAP(OH)YI was further employed by Rodriguez-Gonzalez et al. [100] to build two PROTACs (PROTAC-A and PROTAC-B) with dihydrotestosterone/estradiol to suppress AR and ER in prostate and breast cancer cells, respectively. After 72 h of treatment, both PROTAC-A and PROTAC-B inhibited cell proliferation. PROTAC-B treatment in MCF-7 and T47D cells reduced the expression of cyclin D1 and progesterone receptor (PR) and blocked downstream signaling. Additionally, Rodriguez-Gonzalez et al. [101] created PROTAC-AA by joining a polyarginine tail and two glycine residues to the end of PROTAC-A to improve cell penetration. When compared to the original PROTAC-A, the cells treated with PROTAC-AA indicated at least a five-fold decrease in IC_50_. This approach of improving the permeability of PROTACs and increasing their solubility by adding a polyarginine tail provided a powerful strategy for subsequent PROTAC design.

Cell cycle-related and expression-elevated protein in tumor (CREPT, also known as RPRD1B) is elevated in a variety of cancers. CREPT is an RNA polymerase II-associated protein that promotes transcription of the cell cycle protein cyclin D1 by inducing chromatin loop formation and activating transcription in response to Wnt signaling [102, 103]. The leucine-zipper-like motif is the typical alpha-helix motif used for protein homodimerization and was used as the CREPT binding component [104, 105]. Speltz et al. [106] constructed a PROTAC by linking this sequence to LAP(OH)YI, while adding the membrane penetrating sequence (KRRRR) at the C-terminus. Finally, CREPT was observed after treatment of this PROTAC in Panc-1 cells. The dimerization sequence serves as a novel approach to finding target protein binders and facilitates the development of PROTACs as many proteins cannot be bound by small molecules.

##### (3) ALAPYIP

ALAPYIP is another natural VHL-binding short peptide identified from the HIF1-VHL interaction [58]. It was first utilized by Schneekloth et al. [61] in the construction of PROTACs by joining a ligand for FK506 binding protein (FKBP12) and AR. The PROTACs efficiently drove proteasomal degradation of these two target proteins.

Protein kinase B (PKB or AKT) is a serine/threonine protein kinase that is involved in various cellular processes including cellular metabolism, apoptosis, and cell growth. Aberrant AKT signaling contributes to the occurrence of multiple cancers and diabetes [107]. However, AKT has long been considered a challenging target for drugging. With the assistance of the protein-catalyzed capture agents (PCCs) technique [108], a PCC with a high affinity to Akt was identified and subsequently functionalized with a VHL-binder (ALAPYIP) as well as HIV TAT, resulting in remarkable cell permeability and a high degradation efficacy (over 90%) after 4 h of treatment [109].

Hines et al. [110] developed a phosphorylation-dependent PROTAC (phosphoPROTAC) technique that combines the selective degradation of proteins to the activated state of definite kinase-signaling pathways. The phosphorylation motifs of tropomyosin receptor kinase A (TrkA) and erythroblastosis oncogene B3 (ErbB3) were chosen as the ligands for the recruitment of fibroblast growth factor receptor substrate 2α (FRS2α) and phosphatidylinositol-3-kinase (PI3K), respectively [111, 112]. To create cell-permeable phosphoPROTAC, the VHL recognition peptide ALAPYIP along with TAT was joined to the above specific phosphorylation motif, which would be phosphorylated upon receptor tyrosine kinase (RTK) activation, followed by binding to FRS2α or PI3K and triggering proteasomal destruction. A significant advantage of phosphoPROTAC is that the state of various tyrosine kinase pathways contributes to cell-type-selective degradation. Another strength of this method is that it is less likely to provoke drug resistance. Unlike small-molecule inhibitors that inhibit a full range of kinases, conditional destruction induced by phosphoPROTAC highly relies on the misregulated kinase activity, which attenuates the selective pressure for target kinase mutations.

To achieve specific degradation of β-catenin, a multifunctional protein involved in cell adhesion and the canonical Wnt signaling pathway, Liao et al. [113] first developed and synthesized two distinct β-catenin-specific stapled helical peptides (SAHPA1 and xStAx) with improved membrane permeability and stability using the peptide stapling chemistry. Subsequently, the authors created PROTAC (xStAx-VHLL), a powerful β-catenin degrader, by combining xStAx with the VHL ligand ALAPYIP. This study highlights the potential of peptide-based PROTAC to serve as a new class of drugs that can tackle diseases related to the Wnt/β-catenin signaling by β-catenin degradation.

In addition, a PROTAC, which contained the Tau recognition motif YQYQDATADEQG, a CPP, and the VHL binding motif ALAPYIP was also reported to target the degradation of Tau protein in the mouse brain, [114]; the availability of this multifunctional peptide marks a new era in the treatment of central nervous system diseases.

#### SCF^β−TRCP^-dependent peptide (DRHDS(P)GLDS(P)M) bioTPD

IκBα, a negative regulator of nuclear factor kappa-light-chain enhancer of activated B cells (NF-κB), attaches to SCF^β−TRCP^ upon inflammatory stimulus. The recruitment of IκBα to SCF^β−TRCP^ is largely dependent on a 10-amino acids peptide within IκBα (DRHDSGLDSM), in which two serines can be phosphorylated in response to inflammatory signals. SCF^β−TRCP^ binds to the phosphorylated sequence, triggering subsequent ubiquitination and destruction [115]. Sakamoto et al. [11] constructed a PROTAC (ovalicin-DRHDS(P)GLDS(P)M) using this phosphorylation sequence and demonstrated that this degrader promoted the ubiquitination of METAP2 in vitro. In a later study [12], they constructed more PROTACs targeting ER and AR in the same way and observed apparent degradation of corresponding target proteins in cells through microinjection.

#### KEAP1-dependent peptide (LDPETGEYL) bioTPD

Keap1 is a substrate adaptor protein for the Cullin3 (CUL3)/Ring-Box1 (Rbx1) E3 ubiquitin ligase complex. The transcription factor NF-E2-related factor 2 (Nrf2) is a well-known substrate for Keap1-CUL3. The Nrf2-Keap1 pathway plays a major role in cellular defense against oxidative stress [116]. Lu et al. [117] have identified a short peptide (LDPETGEYL) that could restrain the Keap1-Nrf21 interaction and serve as a strong binder to Keap1. They created a full peptide PROTAC [118] by assembling this Keap1 recognition domain with YQQYQDATADEQG (a Tau-targeting peptide) and poly-D-arginine (CPP). After this PROTAC was applied to different Tau-overexpressing cells including SH-SY5Y, N2a, and PC-12 cells, proteasome-dependent downregulation of intracellular Tau was observed.

#### Proteasome-dependent peptide (RRRG) bioTPD

Bonger et al. [119] created a unique method for protein degradation facilitated by a small molecule Shield-1. The POI was genetically fused with a ligand-induced degradation (LID) domain, resulting in a stable product. Upon the addition of Shield-1, the LID domain rapidly destabilized the targets and induced their degradation. The LID domain contains a 19-amino acid degron appended to the C-terminus of FK506- and rapamycin-binding protein (FKBP). Further truncation experiments revealed that the target protein could be degraded with only a 4-amino acid sequence (RRRG) out of the 19-amino acid degron, and that this could be completely prevented by the proteasome inhibitor MG132. However, the exact degradation mechanism of this domain remains unknown.

The presence of Lewy bodies (LBs) in surviving neurons is a major feature of PD pathogenesis. α-synuclein (α-syn) is the principal component of LBs and is a small 140-amino acid protein that has been highlighted as a major driver of PD pathogenesis [120]. Lysosomal degradation of α-syn by a peptide-based TPD system has previously been documented [24]. However, if the autophagy-lysosomal function is compromised as the disease worsens, clearance of α-syn through the proteasomal pathway would be a good alternative. Qu et al. [121] used the aforementioned short peptide to create a bifunctional degrader. The degrader was comprised of an α-syn binding sequence, RRRG (as a proteasome targeting sequence), and TAT which could induce intracellular α-syn degradation in a concentration- and time-dependent manner [119, 122, 123].

#### Lysosome-dependent peptide bioTPD

##### (1) KFERQ/HSC70

CMA is a type of autophagy specific for substrate proteins containing a pentapeptide motif (KFERQ). The heat shock cognate protein 70 (HSC70) recognizes the KFERQ sequence, forming a substrate/chaperone complex. When the substrate/chaperone complex is proximal to the lysosome, it binds to the extra-membrane region of the lysosome-associated membrane protein type 2 A (LAMP2A), which causes LAMP2A multimerization and ultimately results in substrate protein destruction [124, 125]. KFERQKILDQRFFE, constructed by Fan et al. [24], is formed by linking KFERQ with two other CMA-targeting motifs (CTM), QKILD and QRFFE. They first verified that non-CMA substrate proteins fused with this sequence could be directed to CMA lysosomal degradation. Then they designed a CMA-based degrader bearing three parts: a cell membrane penetrating domain (CMPD), a target protein binding domain (PBD), and a CMA targeting domain (CTM). Fan et al. designed HA-GluN2Bct-CTM, in which GluN2Bct could only bind to the active, but not the inactive form of death-associated protein kinase 1 (DAPK1) [126]. In HEK cells, coexpression of HA-GluN2Bct-CTM with cDAPK1, the active form of DAPK1, resulted in cDAPK1 degradation. Furthermore, a cell-permeable TAT-GluN2Bct-CTM was obtained by the introduction of TAT. After cotreatment of TAT-GluN2Bct-CTM with NMDA (a DAPK1 activator) in HEK cells expressing WT DAPK1, western blot showed that NMDA stimulation promoted WT DAPK1 degradation. To verify the degradation efficacy of this system on other proteins, the investigators further constructed two fusion peptides TAT-βsyn-CTM and TAT-GluN2B9c-CTM for targeting α-syn and postsynaptic density protein 95 (PSD-95). Both peptides in 25 µM could result in the degradation of the corresponding target proteins in neuronal cells.

Cyclin-dependent kinase 5 (CDK5) is a kind of proline-directed serine/threonine kinase and the overactivation of CDK5 has been implicated in neuronal cell death in stroke [127]. Targeted degradation of abnormal CDK5 has a protective effect on injured neurons. Zhou et al. [128] constructed a CDK5-targeted degradation peptide (TAT-CDK5-CTM) using KFERQ and treated cortical neurons (OGD induction) with 5 µM TAT-CDK5-CTM. Significant degradation of CDK5 and a reversal of the damage condition caused by OGD were observed.

##### (2) MDFSGLSLIKLKKQ/The di-leucine sorting signals

Programmed death ligand 1 (PD-L1) is a transmembrane protein that is overexpressed in many types of cancers and is closely associated with immune escape, thus making it a popular target for the oncology landscape [129]. Utilizing the OncoBinder approach [130], Wang et al. [131] identified that huntingtin-interacting protein 1-related protein (HIP1R) interacts with and negatively regulates PD-L1. Of note, they further found that HIP1R targets PD-L1 to lysosomal degradation via a lysosomal sorting signal in HIP1R (966–979). Inspired by the previous work of Fan et al. [24], they constructed this sequence with the PD-L1 binding sequence in HIP1R (784–807) to form a fusion peptide named PD-LYSO. They reported that significant degradation of PD-L1 was observed after overexpressing this fusion peptide in cells. The study revealed that the lysosomal sorting effect of HIP1R (966–979) was due to a di-leucine sorting signal in HIP1R (966–979) that sorts cargos to the lysosome, rather than through the CMA pathway.

##### (3) RGD-integrin-mediated TPD

Recently, Fang et al. [132] first proposed and proved the possibility of RGD-integrin-mediated TPD. They created a bifunctional compound containing a POI-binding ligand and a cyclic RGD peptide as the integrin-binding ligand. The resulting degrader induces the internalization and subsequent degradation of extracellular (NeutrAvidin protein, apolipoprotein E4) or cell membrane proteins (PD-L1) in an integrin- and lysosome-dependent manner. Since α_v_β_3_ integrin is usually overexpressed in many kinds of cancers, this strategy is particularly attractive for the targeted degradation of cancer-relevant proteins. Moreover, Based on the mechanism of receptor-mediated endocytosis and lysosomal degradation, it may be extended to other cell-surface receptors such as the transferrin receptor [133], and folate receptor [134].

#### ClpCP protease-based peptide bioTPD for targeting bacterial proteins

ClpCP protease, a protein-degrading enzyme that recognizes pArg as a degradation tag, is the basis of the BacPROTAC technology. Morreale et al. [8] first designed a class of BacPROTAC degraders using the bacteria ClpCP. BacPROTAC-1, a chimeric small-molecular degrader for monomeric streptavidin (mSA), consists of a pArg derivative (ClpCP ligand) linked to biotin (a high-affinity ligand of mSA) by a linker. BacPROTAC-1 has a high affinity for mSA (K_D_ (dissociation constant) = 3.9 µM) and ClpCP (K_D_ = 2.8 µM), and it can successfully induce the degradation of mSA and three mSA fusion proteins. To improve the poor chemical instability and unfavorable pharmacokinetic profile of the pArg group, Morreale et al. replaced pArg with Cyclomarin A (CymA), a cyclic peptide antibiotic that targets mycobacterial ClpC1 [135] and possesses a pArg-like function. CymA was modified to obtain a high-affinity ClpC1 ligand sCym-1 (K_D_ = 0.81 µM). sCym-1 acts as a ligand for the protease ClpC1 and is linked to JQ1 (an inhibitor of bromodomain-1 (BD1) of BRDT) to form BacPROTAC-3, and the degrader induced the degradation of BRDT_BD1_ in a concentration-dependent manner both outside and inside the bacterium. The emergence of BacPROTACs reveals that bacterial proteins are capable of being selectively degraded through the targeted protease pathway. Identifying ligands of bacterial proteins and linking them to CymA/CymA modifiers to generate ClpCP protease-based BacPROTACs appears to be a feasible strategy for microbial infections.

**Table 2:** Representative example of peptide-based bioTPD

Pathway	Adaptor	Key sequences	POI	POI ligands	Refs
Proteasome	VHL	MLAP(OH)YIPM	METAP2 ER	Fumagillo Estradiol	[60]
		LAP(OH)YI	AR ER CREPT	Dihydrotestosterone Estradiol VRALKQKYEELKKEKESLVDK	[100, 104]
		ALAPYIP	Akt FRS2α PI3K Tau	Recognition peptide for Akt2 (P-ser474) IENPQYFSDA GPGGDYAAMGACPASEQGYEEMRA YQYQDATADEQG	[109, 110, 114]
	β-TRCP	DRHDS(P)GLDS(P)M	METAP2	Ovalicin	[11]
	KEAP1	LDPETGEYL	Tau	YQYQDATADEQG	[118]
	/	RRRG	α-synuclein	GVLYVGSKTR	[136]
Lysosome	/ / /	KFERQKILDQRFFE MDFSGLSLIKLKKQ cyclic RGDyK	DAPK1 α-synuclein PD-L1 PD-L1	A fragment of the GluN2B subunit A short peptide of β-synuclein DKEMAATSAAIEDAVRRIEDMMNQ BMS-8	[24] [131] [132]

Abbreviations: VHL, Von Hippel-Lindau; METAP2, Methionine aminopeptidase 2; ER, Estrogen receptor; AR, Androgen receptor; CREPT, Cell cycle-related and expression-elevated protein in tumor; Akt, Serine/threonine-protein kinase AKT; FRS2α, Factor receptor substrate 2α; PI3K, Phosphatidylinositol-3-kinase; Tau, microtubule-associated protein; α-synuclein, alpha-synuclein; DAPK1, Death associated protein kinase 1; PD-L1, programmed cell death ligand 1

### Fusion protein-based bioTPD

Fusion proteins are a class of complex proteins in which a target protein binding sequence is fused with a full-length or truncated E3 ligase. Unlike traditional PROTAC molecules, which comprise a target protein junction, a linker, and an E3 ligase junction, developers of fusion protein-based degraders genetically engineered their own E3 ligases to change the substrate specificity. The hook effect is an intrinsic property of any TPD molecules that need to form a ternary complex to function. Since the fusion protein-based bioTPD already contains the E3 ligase module, there is no need to form a ternary complex, thereby avoiding the hook effect to a great extent.

**Fig. 2:**
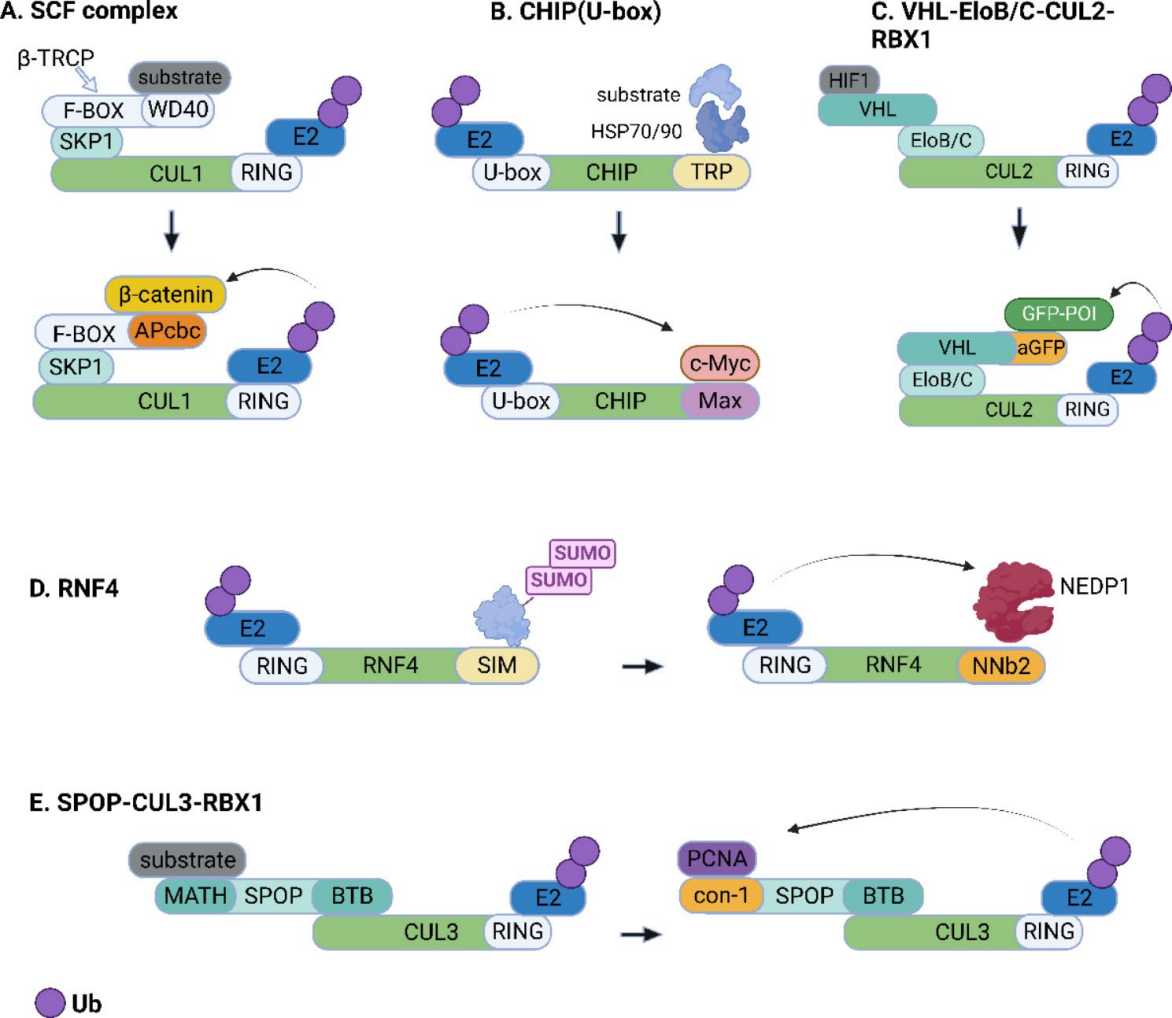
Schematic representation of fusion protein-based bioTPD. (**A**) A β-catenin-binding motif (APcbc) replaces the natural substrate-binding domain of β-TRCP (WD40, etc.) to form a fusion protein for targeted degradation of β-catenin. (**B**) Max (a binding partner of c-Myc) is linked to the U-box of CHIP to form an artificial ubiquitin ligase for targeted destruction of c-Myc. (**C**) An affinity-directed protein missile (AdPROM) system harbors an anti-GFP nanobody (aGFP) that is fused to VHL to recruit any GFP-tagged protein (GFP-POI) to the CUL2 E3 ligase machinery. (**D**) The antibody RING-mediated destruction (ARMeD) system is mediated by a NEDP1-targeting nanobody (NNb2) fused to the RING domain of ubiquitin E3 ligase RNF4 for targeted destruction of NEDP1 via the ubiquitin-proteasome system. (**E**) A high-affinity peptide for PCNA (con-1) replaces the substrate-binding MATH domain of the E3 adaptor SPOP, which enable the ubiquitin tagging of PCNA by the CUL3-based Cullin-RING ligase complex. The figure was created in BioRender.com

To date, more than 600 human E3 ligases have been identified, but only about 10 human E3 ligases have been used to construct classical PROTACs. The development of fusion protein-based bioTPD has facilitated the advancement of additional TPD strategies and applications. The three main types of E3 ubiquitin ligases are known as Really Interesting New Gene (RING), Homologous to the E6-AP Carboxyl Terminus (HECT), and RING-between-RING (RBR) E3s, in which RING E3s are the most abundant types. [39]. Typically, fusion protein-based degraders reported to date hijack a RING-type E3 ligase, including Hsc70-interacting protein (CHIP), Speckle-type POZ protein (SPOP) VHL, RNF4, and SCF^β−TRCP^. In this section, we will describe the development of fusion protein-based bioTPD with different E3 ligases as its backbone (Fig. 2).

#### SKP1-CUL1-F-box-RBX1-based fusion protein

Since the first PROTACs were introduced [11], the structure of PROTAC has been thought to be a fixed mode, comprising a POI binding component, a linker, and an E3 ligase binding component. However, in 2000, Zhou et al. [137] constructed a PROTAC-like fusion protein, and this engineered product also functioned to tether the target protein to an E3 ligase, which prompted the development of fusion protein-based bioTPD.

SCF^β−TRCP^, a common E3 ligase, consists of a combination of Rbx1 (a RING domain), a Cullin1 scaffold, and the F-box protein/SKP1 complex [138], of which the F-box protein is the key component. F-box protein consists of two domains: the F-box domain that binds to SKP1 and the substrate recognition domain (commonly WD40 or leucine-rich repeat sequences to bind different substrate proteins). The yeast-derived F-box protein (Cdc4p) was modified by the investigators and its terminus was attached to the retinoblastoma protein pRB-binding fragment (E7N) to form the Cdc4pF/WD-E7N complex. pRB degradation was observed in yeast cells expressing pRB treated with this engineered protein, exhibiting a half-life of fewer than 60 min. Similarly, the same modification was performed on the human F-box protein β-TRCP and demonstrated similar protein degradation effects. Su et al. [139] performed a similar modification using the F-box protein β-TRCP to selectively eradicate pathogenic β-catenin (Fig. 2A). By analyzing the interacting motifs of β-catenin, a short peptide with 15 amino acids (APCbc) that strongly binds to β-catenin was identified. F3APCbc4 is a fusion protein formed by four APCbc repeat units linked to the F-box structural domain by a linker. Significant reduction of β-catenin and the attenuation of its downstream signal Myc was observed after F3APCbc4 treatment.

To better study intracellular protein function, Caussinus et al. [140] constructed a GFP fusion protein degradation method (deGradFP) using Slmb, a Drosophila melanogaster-derived F-box protein. NSlmb-vhhGFP4 is a recombinant construct formed by linking the F-box domain of Slmb to a nanobody that recognizes GFP (VhhGFP4). Marked reduction of the fluorescent protein H2B-GFP was observed in HeLa S3 cells overexpressing H2B-GFP upon NSlmb-vhhGFP4 expression with minimal off-target effects. The system can target and degrade functional GFP-tagged proteins to mimic the mutagenic loss of proteins, facilitating the study of protein function and corresponding phenotypes.

Baudisch et al. [141] extended the application of the deGradFP method to plant research. The researchers transformed two vectors, NSlmb-vhhGFP4, a targeted degradation peptide, and pGH219, which expresses GFP, together into tobacco plant cells. Western blot showed that GFP degradation by NSlmb-vhhGFP4 was more pronounced compared to the NSnoFbox-vhhGFP4 control group. This study demonstrated for the first time that the fusion protein-based TPD technology can be used to knock out plant POIs via the ubiquitin-proteasome pathway, providing a novel strategy to modulate proteins in crop plants.

#### Ubox (CHIP)-based fusion protein

The RING E3 family has a unique subset known as the U-box that has a RING motif but lacks the Zn^2+^ binding site [39]. Similar to the RING domain, U-box engages E2 and facilitates substrate ubiquitination. CHIP is the most exemplary E3 ligase with a U-box domain.

c-Myc, a proto-oncogene product elevated in malignant tumors, forms a heterodimeric complex with the smaller basic helix-loop-helix/leucine zipper (bHLH/LZ) protein (Max), which contributes to its cancer-promoting functions [142]. Max-U was the first U-box-based fusion protein constructed by Hatakeyama et al. [143], in which Max, as a binding motif for c-Myc, was tethered to the U-box region of CHIP (Fig. 2B). The rational design of Max-U not only verified the interaction between c-Myc and Max, but also enhanced the ubiquitination of c-Myc. Furthermore, targeted destruction of c-Myc protein by the artificial E3 was proven in vitro and in vivo.

Kirsten rat sarcoma viral oncogene homologue (KRAS) is a crucial therapeutic target for pancreatic cancers, lung cancers, and colorectal cancers. Raf-1 acts as a key downstream effector of KRAS, which interacts with KRAS through two key domains, the Ras-binding domain (RBD) and Ras-associated domain (RAD) [144]. On this structural basis, Ma et al. developed a U-box-based fusion protein targeting KRAS for degradation [145]. The engineered E3 ubiquitin ligase, (RBD + CRD)^Raf−1^-U-Box (RC-U), harbors a KRAS recognition motif (RBD + CRD) conformally fused with the charged region and U-box domain of CHIP. A significant reduction in KRAS levels was observed after the transfection of this fusion protein plasmid into PANC-1 cells carrying mutant KRAS.

#### VHL-EloB-EloC-CUL2-RBX1-based fusion protein

VHL is a star E3 ligase in the PROTAC field and numerous scientists have developed a range of peptide fragments and small molecules that bind VHL [74, 76, 146]. However, the discovery of E3 ligase, target protein junctions, and their interaction are often difficult and time-consuming. It is advantageous to be able to rapidly identify the druggability properties of desired proteins in fusion protein TPD. Fulcher et al. [147] established such a functional platform harnessing an engineered VHL E3 ligase and termed it the AdPROM system (Fig. 2C). An anti-GFP nanobody (aGFP) was fused to either the N- or the C-terminus of VHL to establish VHL-aGFP. After transfecting cells with retroviruses encoding VHL-aGFP, endogenous GFP-tagged proteins were degraded via proteasomes. The construction of this platform not only facilitates the study of the function of various proteins but also provides a convenient way to understand the sub-localization of intracellular components. One limitation of this system is that the affinity ligand itself may be recognized as a substrate by the E3. Therefore, the researchers proposed that the AdPROM system can be modified by exclusively replacing the aGFP with smaller binders that bind to specific endogenous proteins. They chose a class of synthetic polypeptides called monobodies [148] that recognizes Src-homology 2 domain-containing phosphatase 2 (SHP2), whose mutations are associated with aberrant Ras/MAPK activation and multiple pathologies, including cancers and Noonan syndrome. Two monobodies, aNSa1 (K_D_ = 14 nM) and aCS3 (K_D_ = 4 nM) that selectively bind to the N-SH2 and C-SH2 domains of SHP2, were ligated to VHL to form VHL-aNSa1 and VHL-aCS3, respectively. A reduction of endogenous SHP2 was observed in cells upon retroviral expression of VHL-aNSa1 or VHL-aCS3. In addition, the Ras/MAPK signaling pathway was also inhibited, observed as a decreased level of ERK1/2 phosphorylation. This study suggests that more affinity ligand options including synthetic monobodies can be exploited for AdPROM-mediated TPD.

#### STUbL RNF4-based fusion protein

RNF4 is a relatively specific E3 ligase, consisting of the C-terminal RING domain which is responsible for its dimerization and recruitment of E2, and the N-terminal domain, which harbors four small ubiquitin-like modifiers (SUMO) interaction patterns (SIM), allowing the E3 ligase to engage SUMOylated substrates. Accordingly, RNF4 is also known as a SUMO-targeted ubiquitin ligase. RNF4 is involved in critical roles in cell growth and DNA damage response as it is a regulator of those SUMOylated proteins, including breast cancer type 1 susceptibility protein (BRCA1) and DNA damage checkpoint protein-1 (MDC1) [149, 150].

Ibrahim et al. [151] constructed an antibody RING-mediated destruction (ARMeD) system in which the SUMO recognition domain of RNF4 was replaced with a substrate-specific-nanobody. The anti-GFP nanobody (GNB) was used as a model nanobody and was tethered to either one or two RING domains of RNF4, generating GNB-1×RING and GNB-2×RING. Initially, two yellow fluorescent protein (YFP) fusion proteins (YFP-PARG and YFP-PML), which could be theoretically recognized by GNB were efficiently depleted in cells expressing the doxycycline (Dox)-inducible GNB-RING constructs. Subsequently, nanobodies (NNb2 and NNb9) targeting endogenous NEDD8 specific protease (NEDP1) were tethered to the RING of RNF4 to form Dox-inducible NNb-RING fusions (Fig. 2D). Apparent degradation of the NEDP1 and accumulation of NEDD8 and its dimer were observed under the same condition. Of note, proteomic analysis revealed that no observable off-target destruction occurred, indicating that the high-affinity nanobody ensured a high selectivity of the ARMeD system. To circumvent genetic manipulation and Dox induction, the researchers introduced the recombinant nanobody-RING fusion into cells by electroporation and surprisingly found that the elimination of endogenous target proteins occurred within minutes. This transient and rapid degradation method can be used to study rapid-changing cellular processes like the cell cycle.

#### SPOP-CUL3-RBX1-based fusion protein

Motivated by the deGradFP technique established by Caussinus et al. [140], Shin et al. [152] hypothesized that optimizing the E3 architecture might achieve a better degradation effect. They constructed various synthetic E3 ligase candidates in which the adaptor protein of distinct E3 was fused to an anti-GFP nanobody (vhhGFP4). They found that vhhGFP4-SPOP (Ab-SPOP), in which vhhGFP4 was joined to SPOP (an adaptor protein of the CUL3-RING E3 ligase), manifested optimal clearance of H2B-GFP in cells, even compared with the deGradFP system.

Similarly, Lim et al. [153] also carried out a systematic study by constructing a panel of synthetic E3 ligases targeting GFP, which they termed bioPROTACs (biological PROTACs). Their degradation effects in HEK 293 Tet-On 3G cells were validated. Seven GFP binders (nanobodies: vhhGFP4, DARPin: 3G86, αReps: bGFP-A, bGFP-C, and three monobodies: GS2, GL6, GL8), and ten different E3 ligases (βTRCP, FBW7, SKP2, VHL, SPOP, CRBN, DDB2, SOCS2, ASB1, CHIP) were used to explore the flexibility of constructing a bioPROTAC. Except for the two weak binders, 5 of 7 GFP binders were able to degrade GFP despite the distinct diversity in structure, size, and binding affinity. Additionally, the majority of vhhGFP4-E3 fusions can degrade GFP, with SPOP displaying the greatest efficacy. Indeed, 8 of 10 mammalian E3 ligases displayed remarkable degradation activities. Furthermore, they tested the degradability of endogenous proliferating cell nuclear antigen (PCNA, an auxiliary protein of DNA polymerase δ [154]) via the proteasomal pathway using bioPROTAC. As expected, the rationally-designed SPOP-con1, a product in which the BTB domain of SPOP was fused to con-1 (a binding motif of PCNA), could induce PCNA degradation efficiently (Fig. 2E).

In 2021, Lim et al. [155] further generated a series of bioPROTACs against KRAS-GFP. As mentioned previously, SPOP was found to be the most suitable E3 ligase and was validated for use in RAS degradation by linking it to four high-affinity RAS binders (NS1, K27, K55, R11.1.6). Compared to the other three bio-degraders, SPOP-K27 showed complete pan-RAS degradation efficiency, and additionally degraded mutant KRAS^G12D^ protein and inhibited the proliferation of KRAS-mutant AsPC-1 cells.

Overall, bioPROTACs serve as a powerful tool for interrogating target biology, druggability, and additional approaches toward the creation of TPD degraders. However, despite these broad advantages, fusion protein-based bio-degraders are highly polar and lack membrane permeability and bioavailability. Accordingly, they require external means to facilitate their entry into cells (e.g. transfection, electroporation, membrane permeable peptides). Under this circumstance, fusion proteins could serve as an important complementary technology for screening a suitable E3, examining the druggability of POIs, and laying the foundation for the advancement of PROTAC. With the help of more advanced gene delivery systems, fusion protein-based bioTPD is a promising candidate for more direct application in clinical use.

### Antibody (fragments)-based bioTPD

#### TRIM-Away

**Fig. 3:**
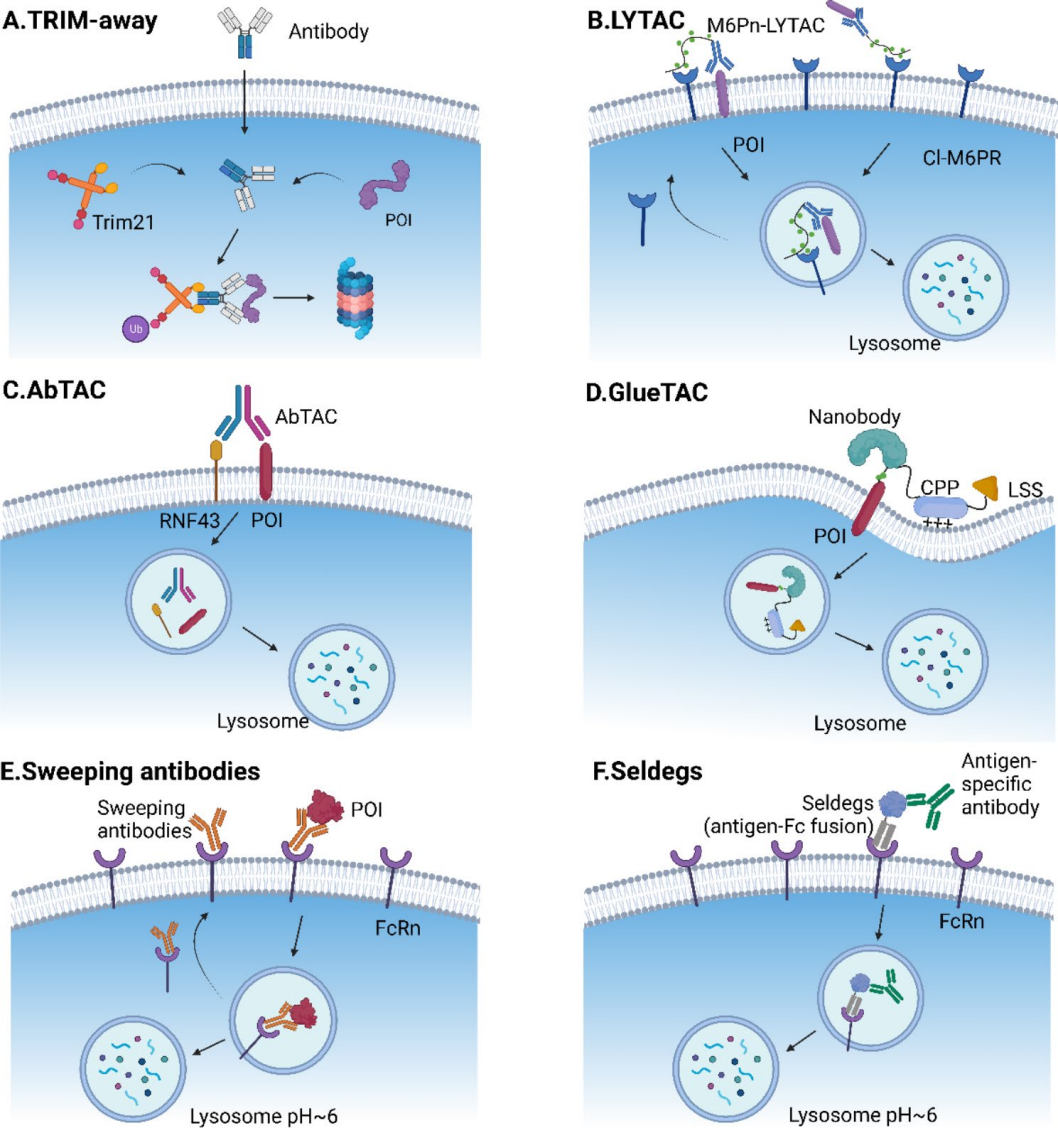
Schematic representation of antibody-based bioTPD. (**A**) Trim21 recognizes the Fc domain of antibodies and is auto-ubiquitinated. Ubiquitinated Trim21 and its antibody/protein complexes are targeted for proteasomal degradation. (**B**) LYTAC is composed of a small molecule or an antibody coupled to a ligand that binds to LTRs, such as CI-M6PR and ASGPR. The LYTAC-POI complex is endocytosed along with LTR, followed by lysosomal degradation. (**C**) AbTAC, a bispecific antibody, concurrently recruits E3 ubiquitin ligases RNF43 and a membrane POI. The POI is degraded in a lysosomal-dependent manner. (**D**) GlueTAC consists of a covalent nanobody for POI targeting, a CPP for rapid endocytosis, and a lysosome-sorting sequence (LSS) for lysosomal degradation. (**E**) A sweeping antibody is an IgG that is engineered to connect to the neonatal Fc receptor (FcRn) at both neutral and acidic pH and a secreted POI only at neutral pH. The FcRn transports the POI-antibody-FcRn complex to the endosome. In the acidic environment, the POI leaves the sweeping antibody and proceeds to the lysosome, while the remained antibody-FcRn recycles back to the cell membrane to catch more targets (**F**) Seldegs are engineered Fc-antigen fusions with the capability to capture circulating antibodies and bring them to lysosomal degradation. The figure was created in BioRender.com

TRIM21, an E3 ligase, natively recognizes the Fc fragment of an antibody and subsequently drives the antibody-POI complex or antibody-bound pathogens to the proteasome [156, 157]. Trim-Away is an antibody-based bioTPD technology developed in 2017 that exploits commercially-available antibodies and TRIM21 for rapid protein disruption (Fig. 3A) [16]. The endogenous level of TRIM21 is sufficient for protein degradation in several cell types, such as primary cells. If insufficient, TRIM21 needs to be exogenously delivered together with the antibody by co-electroporation or microinjection. The proof of concept of Trim-Away was verified using a range of 9 endogenous proteins in 10 cell types, demonstrating the widespread application and substrate independence. Strikingly, the degradation process is acute and rapid within minutes. Since Trim-Away utilizes antibodies, a wide range of POIs with commercially-available antibodies combined with TRIM21 are available for functional studies. However, an apparent drawback to Trim-Away is that it is difficult for antibodies to cross the cell nucleus and membrane without external assistance. Clift et al. [16] further illustrated that Fc-nanobody fusion was compatible with Trim-Away for the degradation of nucleus proteins. In 2021, Chen et al. [158] constructed a novel Trim-Away system termed TRIMbody by fusing a POI-binding nanobody with the RBCC motif of TRIM21 to avoid microinjection or electroporation. The inducible expression of EGFP TRIMbody efficiently degraded EGFP in HEK293T-EGFP cells via both the proteasome and autophagy-lysosome pathways. Recently, two papers [159, 160] reported a BCL11A biological degrader independently and performed a proof-of-concept study based on the Trim-Away strategy. They produced plasmids of their nanobodies fused to Trim21 or Fc and performed lentiviral transduction to confirm the selective degradation of BCL11A. Laura M. K. Dassamaf [160]further designed a cell-permeant fusion of their nanobody to a cell-permeant miniature protein (ZF5.3) and an E3 adaptor (SPOP or RNF4). The fusion was expressed and showed efficient depletion of cellular BCL11A. This strategy can be employed for the creation of more cell-permeant protein-based degraders.

The practical application of TRIM-Away has been adopted in a variety of cell types and in vivo embryo development [128, 161–165]. Unlike clustered regularly interspaced short palindrome repeats (CRISPR)/Cas9 and RNA interference technologies, Trim-Away can directly degrade specific proteins within any cell type. Moreover, Trim-Away can also differentiate between different splice or mutant protein variants, and post-translationally modified proteins, which might open new avenues for disease research.

#### LYTAC

Membrane-associated and extracellular proteins, which account for the products of 40% of all the encoding genes [166], play a vital role in cancers, autoimmune disorders, and neurodegenerative diseases [167]. LYTAC is a superior complement to PROTACs since it selectively targets these proteins to lysosomal degradation.

Previous studies have demonstrated that lysosome-targeting receptors (LTRs) residing on the cell surface facilitate the intracellular transport of proteins to lysosomes [168]. This process was harnessed to generate the first LYTAC, which consists of an antibody connected with an LTRs-bounding ligand. The first reported LYTAC was based on the cation-independent mannose-6-phosphate receptor (CI-MPR, IGF2R) which serves as a lysosome shuttle [17]. The antibody was directed against the POI, while the conjugated multiple serine-O-mannose-6-phosphonate (M6Pn) residues interacted with CI-MPR for internalization via clathrin-mediated endocytosis, and the POI and LYTAC were subsequently dragged to the lysosome for degradation (Fig. 3B). This LYTAC platform has shown great promise in the degradation of plasma proteins (apolipoprotein E4) and multiple membrane proteins, including EGFR, transferrin receptor-1 (TfR/CD71), and PD-L1.

LYTAC that is engaged in tissue-specific LTRs offers the opportunity to induce POI degradation in specific tissues. Unlike CI-M6PR, which is ubiquitous, some LTRs are tissue-specific. For example, the asialoglycoprotein receptor (ASGPR) is a well-defined LTR primarily expressed in hepatocytes with 500,000 copies per cell [169]. The ASGPR-based LYTAC (GalNAc-LYTACs) is generated by the fusion of antibodies or peptides with N-acetyl galactosamine (GalNAc) or tri-GalNAc as ASGPR ligands [18, 170, 171]. In liver cancer cells, different GalNAc-LYTACs have been shown to downregulate EGFR and integrins. In addition, GalNAc-LYTACs are superior in internalizing extracellular components compared to M6Pn-LYTACs in HEPG2 cells, which is likely due to the high level of ASGPR over CI-M6PR in hepatocytes [18]. With the preliminary success of CI-MPR- and ASGPR-based LYTAC, it will be promising to exploit other cell-specific and tissue-specific LTRs [10]. Importantly, it is worth noting that current LYTACs are degraded along with POIs, which suggests a lack of desirable catalytic function compared with most PROTAC degraders [45]. In addition, the large molecular weights of the antibodies and immune responses induced by the conjugated glycopeptide should be addressed.

#### AbTAC

Bispecific antibodies are recombinant antibodies that can recognize two different antigens or epitopes. Bispecific antibodies are a rapidly growing research area in the field of cancer immunotherapy [172]. In 2021, Wells’ group [19] utilized a bispecific antibody, termed AbTAC, to concurrently recruit E3 ubiquitin ligases RNF43 and PD-L1. RNF43 is a transmembrane E3 ligase with an intracellular RING domain and a structured ectodomain [173]. The AbTAC was constructed by fusion of two half IgGs targeted for PD-L1 and the ectodomain of RNF43 (Fig. 3C). Biolayer Interferometry (BLI) experiments confirmed a high affinity to both antigens. Importantly, the AbTAC achieved efficient depletion of PD-L1, with a half-maximal degradation concentration (DC_50_) of 3.4 nM and a maximum degradation efficacy of 63% at 24 h in MDA-MB-231 cells. Unexpectedly, the AbTAC depleted PD-L1 in a lysosomal-dependent manner, rather than a proteasomal-dependent manner, which is closer to LYTAC. The exact mechanism of action of AbTAC should be explored in future studies. Similar to LYTACs, no large cellular proteomic perturbations occurred following AbTAC treatment. Recently, Wells’ group [174] generated a new AbTAC system by co-opting another transmembrane E3 ligase zinc and ring finger 3 (ZNRF3) to disrupt EGFR and PD-L1. Furthermore, they illustrated that the antibody binding epitopes on the E3 ligase and the POI were of greater importance than the affinities of AbTAC. Recently, a similar approach was reported in which bispecific proteolysis-targeting antibodies (PROTAB) that tether cell-surface E3 ubiquitin ligases (RNF43, ZNRF3) to transmembrane proteins (insulin growth factor 1 receptor (IGF1R)) [175]. The PROTAB induces target internalization and degradation of IGF1R in a ligase-dependent manner. The study also demonstrated the generality of this PROTAB platform on the degradation of human epidermal growth factor receptor 2 (HER2) and PD-L1. Of note, given that RNF43 and ZNRF3 are downstream of Wnt signaling, the PROTAB strategy can enable Wnt-hyperactivated tumors targeting and specific degradation of cell-surface proteins.

Despite the rapid development of proteasome-based TPD technology, only cytosolic E3 ligases have been used up to this point. The above studies were the first to extend proteasomal degradation to cell-surface E3 ligases, offering more complementary methods for the targeted degradation of membrane-bound proteins.

#### GlueTAC

Unlike other antibody-based TPD technologies (e.g. LYTAC, AbTAC), Zhang et al. [20] developed another method for targeted degradation of cell-surface proteins based on covalent nanobody-PROTAC (GlueTAC). They first screened a PD-L1-targeted covalent nanobody variant (Gluebody) using the MS-assisted screening platform (MSSP) in combination with the genetic code expansion (GCE) strategy. The covalent nanobody not only contributes to better cell penetration but also a higher binding affinity and lower off-target effects due to the covalent interaction between the nanobody and the POI. Afterward, the GlueTAC was coupled to a CPP (GGGRRRRRRRRR) and the lysosome-sorting sequence (NPGY), allowing rapid endocytosis and lysosomal degradation [176, 177] (Fig. 3D). Ultimately, the rationally-designed GlueTAC achieved efficient degradation of cellular PD-L1 and demonstrated superior antitumor activity in the PD-L1-EGFP/A375 tumor model even compared with Atezolizumab, an FDA-approved anti-PD-L1 antibody.

Compared with LYTAC or AbTAC, GlueTAC represents a universal membrane protein targeted degradation strategy as it is cell-type-independent and receptor/E3 ligase-independent. However, safety concerns originating from the introduction of unnatural amino acids and pharmacokinetic profiles of nanobodies should be considered in further studies.

#### Sweeping antibodies

The sweeping antibody is a recyclable degrader that specifically targets extracellular antigen degradation. With the recycling property, it can reduce the dose and frequency of administration of traditional antibodies [178]. The sweeping antibody is a pH-dependent bispecific IgG that is engineered to bind to the neonatal Fc receptor (FcRn, a recycling receptor) at a neutral/acidic pH and secreted/soluble proteins only at a neutral pH. FcRn, a specific membrane receptor for IgG and albumin, plays a central role in prolonging the lifespan and dynamic balance of these proteins [179, 180]. The FcRn transports the POI-antibody-FcRn complex to the endosome. In the acidic environment, the POI leaves the sweeping antibody and proceeds to the lysosome, while the remaining antibody-FcRn recycles back to the cell membrane to catch more targets [172] (Fig. 3E).

Igawa et al. [22] first constructed a sweeping antibody by manipulating the variable region of the antibody to enable pH-dependent binding and modifying the constant region to improve its affinity to FcRn to facilitate internalization. They showed that an anti-interleukin-6 receptor (IL-6R) sweeping antibody derived from tocilizumab cleared plasma IL-6R 50- to 1000-fold in mice in comparison with a conventional antibody. Inspired by this technology, Muramatsu et al. [181] designed myostatin-specific sweeping antibodies aiming to reinforce muscle strength by sweeping the latent form of myostatin. Similarly, Sampei et al. [182] engineered a pH-dependent antibody specific to complement component 5 (C5) and demonstrated a long-lasting clearing activity of C5 in cynomolgus monkeys, suggesting a promising application of sweeping antibodies in modulating the disordered complement system.

#### Seldegs

Seldegs are engineered antibody fragment-antigen fusion proteins designed for selective depletion of endogenous antigen-specific immunoglobulin G (IgG) based on FcRn-IgG interactions. Such clearing agents offer promising avenues in therapeutic areas such as antibody-mediated autoimmunity disorders, transplant rejection, and the clearance of IgG-drug complexes [183].

Similar to sweeping antibodies, Seldegs are mainly comprised of an engineered Fc domain targeting FcRn. Seldegs were developed from the earlier discovery of Abdegs. In 2015, Ward’s group [183] first engineered the Fc fragment of a human IgG to increase affinity as well as mitigate pH dependence on FcRn. The mutated IgG was named Abdegs (antibodies that enhance IgG degradation) and induced rapid clearance of unmanipulated circulating IgG concentration in vivo. As Abdegs nonspecifically induced degradation of all circulating IgGs, they next generated a novel antibody-based clearing agent with high selectivity in 2017 termed Seldegs (selective degradation) [23]. Seldegs are Fc-antigen fusion proteins with the capability of capturing circulating antibodies and targeting them for lysosomal degradation based on high pH-independent interactions between Seldegs and FcRn (Fig. 3F). Specific mutations were introduced into the Fc domain to ablate affinity for FcγRs and increase affinity for FcRn [184]. Two recombinant antigens, myelin oligodendrocyte glycoprotein (MOG) and HER2 were fused with the mutated Fc domain. As a consequence, the MOG- and HER2-Seldeg induced lysosomal delivery of corresponding antibodies, anti-MOG and anti-HER2 antibodies in FcRn-expressing cells, and induced in vivo clearance of targeted antibodies at a relatively low dose in contrast to Abdegs or earlier FcRn antagonists with no effect on total IgG level [183, 185, 186]. Furthermore, their subsequent investigation verified that MOG-Seldeg treatment specifically removed patient-derived MOG antibodies, which ameliorated the symptoms of autoimmune encephalomyelitis in mice [187]. Notably, since Seldegs comprise a recombinant antigen, it should be ensured that the selected antigen only binds to the targeted autoantibodies, making no alteration to antibodies of non-targets [188].

### Nucleic acid-based bioTPD

Proteins with catalytic activity tend to be relatively druggable. However, many other protein families such as RNA binding proteins (RBPs) and transcription factors (TFs) remain intractable due to their lack of binding sites. The discovery of specific binding ligands is critical to develop drugs targeting such proteins. Nucleic acids can bind to specific protein domains and are thus powerful biomacromolecule ligands for creating degraders. Nucleic acid-based degraders have evolved rapidly since 2021 and in less than one year, TF PROTACs, RNA-PROTAC, G-quadruplex (G4)-PROTAC, and aptamer-based PROTAC have been developed. Their emergence provides the possibility to directly target diseases caused by RBPs, TFs, or G4-binding proteins.

#### RNA-PROTACs

**Fig. 4:**
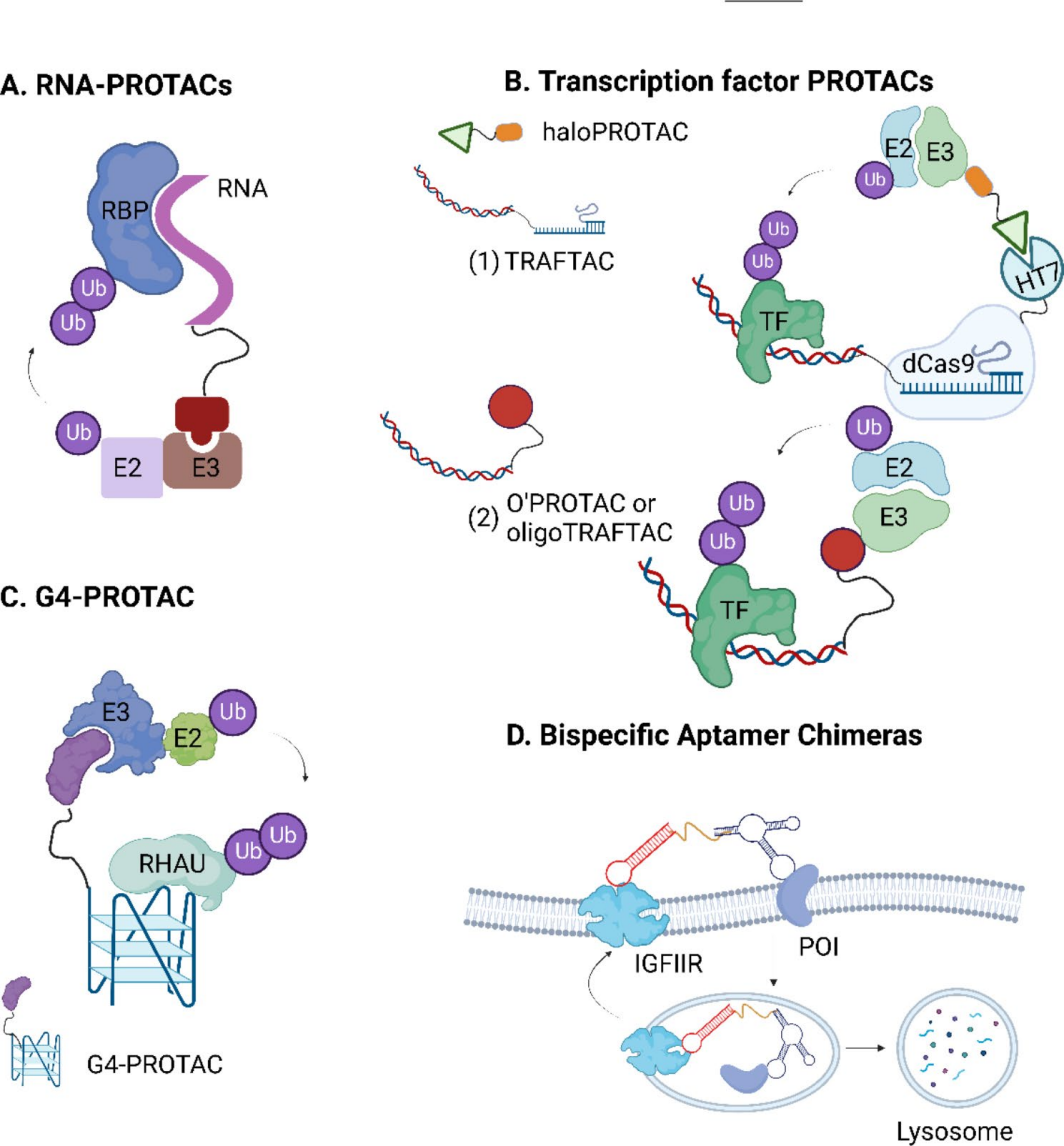
Schematic representation of nucleic acid-based bioTPD. (**A**) RNA-PROTAC consists of a short oligonucleotide that is iso-sequential with the RNA consensus binding element as an RBP-recognizing ligand and an E3-recruiting peptide for proteasomal degradation. (**B**) TRAFTAC (1) is a bifunctional chimeric oligonucleotide (dsDNA-CRISPR-RNA) that binds to the transcription factor with an oligonucleotide and recruits E3 ligases through dCas9-HT7 fusion protein in the presence of a haloPROTAC. O’PROTAC or OligoTRAFTAC (2) contains a double-stranded oligonucleotide as a transcription factor-recognizing ligand and a VHL-recruiting moiety. (**C**) G4-PROTAC uses G4 as a warhead of the PROTAC for targeted degradation of a G4-binding protein RHAU (a DEAH-box helicase). (**D**) Bispecific aptamer chimeras utilize DNA aptamers to target the POI and lysosome-shuttling receptor IGFIIR, respectively. The figure was created in BioRender.com

RBPs constitute a large class of over 2,000 proteins that interact with transcripts in most RNA-driven processes [189]. RBPs bind to RNAs in a dynamic and sequence-dependent manner to form ribonucleoprotein (RNP) complexes and coordinate RNA processing [190, 191] (Fig. 4A). Genetic alterations in RNA-binding proteins can lead to genetic diseases, including amyotrophic lateral sclerosis caused by fused-in-sarcoma (FUS)/TAR DNA binding protein-43 (TDP-43) mutations, and myelodysplastic syndromes caused by U2AF35/ splicing factor 3b subunit 1 (SF3B1) mutations [192]. However, most RBPs are undruggable by conventional therapies or small-molecule PROTACs [193].

In 2021, Ghidini et al. [194] introduced a novel class of chimeric structures, termed RNA-PROTAC, which aims to degrade RBPs. RNA-PROTAC uses short oligonucleotides that are iso-sequential with the RNA consensus binding element of an RBP as an RBP-recognizing ligand and links it to an E3-recruiting peptide. The studies confirmed a proof-of-concept for RNA-PROTAC by targeting two RBPs, a stem cell factor LIN28 and a splicing factor RBFOX1. The rationally-designed chimeras selectively degraded two RBPs in cancer cell lines in a ubiquitin-dependent manner.

#### Transcription factor PROTACs

TFs are DNA-binding proteins that directly or indirectly regulate gene expression and their dysfunction causes many pathologies, including cancers [195]. As TFs lack the active sites or allosteric sites commonly found in kinases or enzymes, it is difficult to design TF-binding small-molecule inhibitors, making TFs poorly druggable targets.

Harnessing the intrinsic TF DNA-binding ability, Crews’ group [196] developed the TRAnscription Factor Targeting Chimeras (TRAFTACs) technology to induce TFs degradation. TRAFTACs are bifunctional chimeric oligos (dsDNA-CRISPR-RNA) that bridge the TF of interest (TOI) with an ectopically expressed dCas9-Halotag7 fusion protein (dCas9HT7) to form a complex. Incubation with a haloPROTAC recruits the VHL-E3 ligase to the complexed fusion protein and then induces TOI deconstruction (Fig. 4B). The TRAFTAC system has successfully degraded two oncogenic TOIs, NF-κB and brachyury via the proteasomal degradation pathway. The researchers further demonstrated that TRAFTACs could degrade zebrafish brachyury and induce a no-tail phenotype, suggesting a therapeutic potential to degrade disease-relevant TFs. Later, Crews’ group [194] further developed the second generation of TRAFTACs technology called oligoTRAFTAC. The oligoTRAFTAC system succinctly consists of a TF binding oligonucleotide and an E3 ligase-recruiting ligand without the requirement for genetic modification (Fig. 4B). Two specifically-designed oligoTRAFTACs effectively degraded c-Myc and brachyury in cells and zebrafish.

Meanwhile, Shao et al. [197] reported a similar oligonucleotide PROTAC structure termed O’PROTACs. The O’PROTACs system also contains a double-stranded oligonucleotide as a TF-recognizing ligand and a VHL-recruiting moiety (Fig. 4B). The degraders successfully promoted the degradation of two oncogenic TFs, lymphoid enhancer-binding factor 1 (LEF1) and ETS-related gene (ERG), and showed suppressive effects in prostate cancer modes. Compared with the first-generation TRAFTACs, oligoTRAFTAC and O’PROTAC exclude the artificially engineered dCas9-HT7 fusion protein which simplifies the synthesis process and improves the limitation of nucleic acid-based bioTPD in clinical application.

Different from oligoTRAFTACs and O’PROTACs structure, Liu et al. [198] used a click reaction to connect DNA oligonucleotides to E3 ligase ligands. They synthesized and optimized two series of VHL-based TF-PROTAC (dNF-κB and dE2F) by changing the length and structure of the linker via a copper-free strain-promoted azide-alkyne cycloaddition (SPAAC) reaction. They validated that the two TF-PROTACs selectively degraded p65 and E2F1 protein in cells, respectively, and exhibited promising antiproliferative effects.

Compared with RNA-PROTACs, the DNA-based PROTACs are relatively more stable. Additionally, the DNA binding specificity to TFs is better than the binding specificity of RNA to RBPs [198].

#### G4-PROTAC

G4 binding proteins are involved in important biological processes, such as telomere maintenance, DNA replication, and gene transcription [199]. The abnormality of G4 binding proteins also is associated with various human diseases, such as cancers and amyotrophic lateral sclerosis (ALS) [200]. G4 are four-stranded DNA secondary structures with rich guanine sequences [200].

Patil et al. [201] first used G4 as a warhead of PROTAC for targeted degradation of a G4-binding protein (DEAH-box helicase RHAU) (Fig. 4C). RHAU is overexpressed in tissues from patients with C9orf72-related ALS and is, therefore, a vital target for ALS treatment [202]. An RHAU-bound all-parallel-stranded G4 (sequence TT(GGGT)_4_) was linked to two distinct E3 ligands, CRBN and VHL, via the click reaction. Both G4-PROTAC displayed potent and specific degradation of RHAU in HeLa and K-562 cell lines. Accordingly, the study highlights the feasibility of designing a TPD construct using a non-canonical nucleic acid structure and offers an alternative therapeutics toolbox against diseases caused by G4-binding proteins.

#### Bispecific aptamer chimeras

Aptamers are short single-stranded oligonucleotides (ssDNA or ssRNA) that can selectively bind to protein targets or peptides with high affinity, either in their native states or on cellular membranes [203]. Aptamers can be theoretically screened in vitro by a selection strategy called systematic evolution of ligands by exponential enrichment (SELEX), which broadens the target range of aptamers to all accessible proteins [204–206]. Thus, nucleic acid ligands can be effectively utilized to target proteins for proteasomal and lysosomal degradation.

Inspired by the design concept of LYTAC that allows cell-surface lysosome-shuttling receptors to direct a membrane protein to the lysosomes for degradation [17], Miao et al. [21] designed the first bispecific aptamer conjugates simultaneously targeting Insulin-like growth factor type II receptor (IGFIIR, a lysosome-shuttling receptor) and the membrane POI (Fig. 4D). They verified that the chimeras successfully shuttled two membrane proteins, mesenchymal epithelial transition (Met) and tyrosine protein kinase-like 7 (PTK-7) to the lysosomes, and rapidly and efficiently degraded them at nanomolar concentrations. Benefiting from the development of SELEX/Cell-SELEX for selected aptamers [206, 207], this technology might allow more membrane protein-associated degradation than LYTAC. However, the stability and off-target effects of such aptamer chimeras need to be elucidated in further studies [208].

## Options for improving the delivery efficacy of bioTPD

From Table 1, it is clear that poor cell permeability, unfavorable pharmacokinetic performance, low stability as well as low delivery efficacy are common limitations for most bio-degraders. Although bioTPD has the potential to generate alternative therapeutic agents with high specificity, delivery, and degradation efficacy are key hurdles to be addressed.

### Utilization of CPP

The CPPs, including oligoarginine (RRRRRRRR), HIV-1 Tat peptide (YGRKKRRQRRR), pentapeptide (RRRRK), and Xentry (LCLRPVG) have already been used to facilitate cell entry through direct penetration or endocytosis, which are canonical avenues for delivery of different cargos, including peptides, oligonucleotides, proteins, and nanocarriers [104, 110, 209–211]. Several studies have utilized these CPPs to improve the cell permeability of bioTPDs. For example, a poly-D-arginine motif was incorporated at the end of two phosphoPROTACs that couple the tyrosine phosphorylation sequence with a VHL-recruiting peptide to permit cell permeability [110]. A similar result was shown in TAT-modified hydrophobic tags which conjugated peptides for disruption of TAR DNA binding protein 43 (TDP-43) and finally reduced the cytotoxicity induced by TDP-43 in N2a cells [210]. Additionally, it was evident that the introduction of RRRRK sequence also enhanced the cell permeability of CREPT-targeted PROTAC in AsPc-1 and MIA PaCa-2 cells, which was comparable with the action of TAT [104]. In addition to peptide-based TPDs, CPPs have also been used to facilitate the cell entry of antibody-based cargo. GlueTAC, as mentioned before, is such a paradigm. To ameliorate the cell entry and proteolytic capabilities of the glueTAC, a CPP peptide consisting of nine D-arginines was tethered between the C-terminal of an anti-PD-L1 nanobody and the N-terminal of a lysosomal sorting sequence [20].

### Improve selectivity by active targeting ligands

Selectivity to the target POI is crucial for any therapeutic candidate. Candidates with high selectivity can reduce off-target effects and thus prevent latent side effects in clinical trials. Improving selectivity is a constant challenge for the development of TPD technology.

The selection of E3 ligases is important to improve selectivity because the natural expression of E3 ligases varies remarkably in different tissues, cells, and subcellular compartments [212]. Additionally, several studies have demonstrated that by optimizing the linker length and stabilizing the ternary complexes, PROTAC moieties can enhance selectivity to their POIs beyond their parent ligands [213]. However, it has been quite challenging to computationally predict the optimal linkers and lengths without the POI-PROTAC-E3 ternary complex structures [212].

Another attractive strategy to enhance TPD selectivity is by endowing these degraders with active targeting capabilities. Through connection to an active targeting ligand, such as an antibody or aptamer, the degraders can potentially target a wide variety of tissues or specific cell types. Antibody-PROTAC conjugates, also termed Ab-PROTAC, which are analogs of antibody-drug conjugates (ADC) have emerged in the past two years. In two examples, an anti-HER2 antibody was joined to a BRD4 or estrogen receptor alpha (ERα) degrader [214, 215]. Accordingly, these Ab-PROTACs showed specific internalization and importantly, displayed strong POI degradation activity in HER2-positive cells. Dragovich et al. [216, 217] systemically constructed a series of antibody-PROTAC conjugates tethering a BRD4 degrader to anti-STEAP1 or anti-CLL1 antibodies, and systemically demonstrated that the linker between the antibody and PROTAC and its cleavable property played an important role in the degradation efficacy.

Considering that aptamers are known as ‘chemical antibodies’, the aptamer-PROTAC conjugate was recently developed by conjugating a bromodomain and extra-terminal (BET) degrader to the nucleic acid aptamer AS1411 via a GSH-responsive linker [218]. The aptamer, a transport agent for cell-surface nucleolin-expressing cancer cells, significantly improved degrader uptake and internalization in nucleolin-overexpressing MCF-7 cells, leading to high efficiency in vivo BRD4 degradation and antitumor potency as well as decreased toxicity. Thus, aptamer conjugation might be an advantageous option for PROTAC delivery, similar to antibody conjugation. Overall, these studies demonstrate proof-of-concept for tissue/cell-specific target degradation, overcoming constraints of PROTAC selectivity, with significant potential for application to other types of TPD, including bioTPD.

### Drug delivery systems

Encouragingly, we have witnessed rapid advancements in drug delivery systems in the past two decades. Such systems have offered unique advantages in delivering traditional therapeutic agents such as small molecules, genes, and proteins that may have pharmacologically undesirable properties. Benefiting from their broad loading capacity, improved pharmacokinetics/pharmacodynamics performance, accelerated cellular uptake, and multifunctional modification, drug delivery nanocarriers may also play a vital role in the development of bioTPD for clinical translation through maximizing their efficacy and overcoming their limitations [219].

In recent years, researchers have begun to utilize drug delivery systems to deliver small-molecule chimeric degraders as well as bioTPD components to improve their potential in vivo applications. As for small-molecule PROTACs, there have been several nanoformulations, including polymeric nanoparticles [219, 220], lipid-based nanoparticles [221], biomimetic nanocarriers [222], and two-dimensional nanocarriers [223] reported to improve their destruction efficacy. For example, PLGA-PEG nanoparticles were fabricated to encapsulate hydrophobic ARV-825 and finally achieved passive targeting and enhanced antitumor effect in vivo [220]. An ARV-825-loading substance P (SP) peptide-modified polymeric micelles promoted ARV-825 movement across the blood-brain barrier and displayed outstanding anti-proliferative effects against glioma [219], opening an avenue for glioma therapy harnessing TPD technology. Collectively, it can be stated that existing delivery systems are quite suitable for the delivery of small-molecule TPD. As for bioTPD, different types of bioTPD have different delivery demands due to their natural properties.

**Fig. 5:**
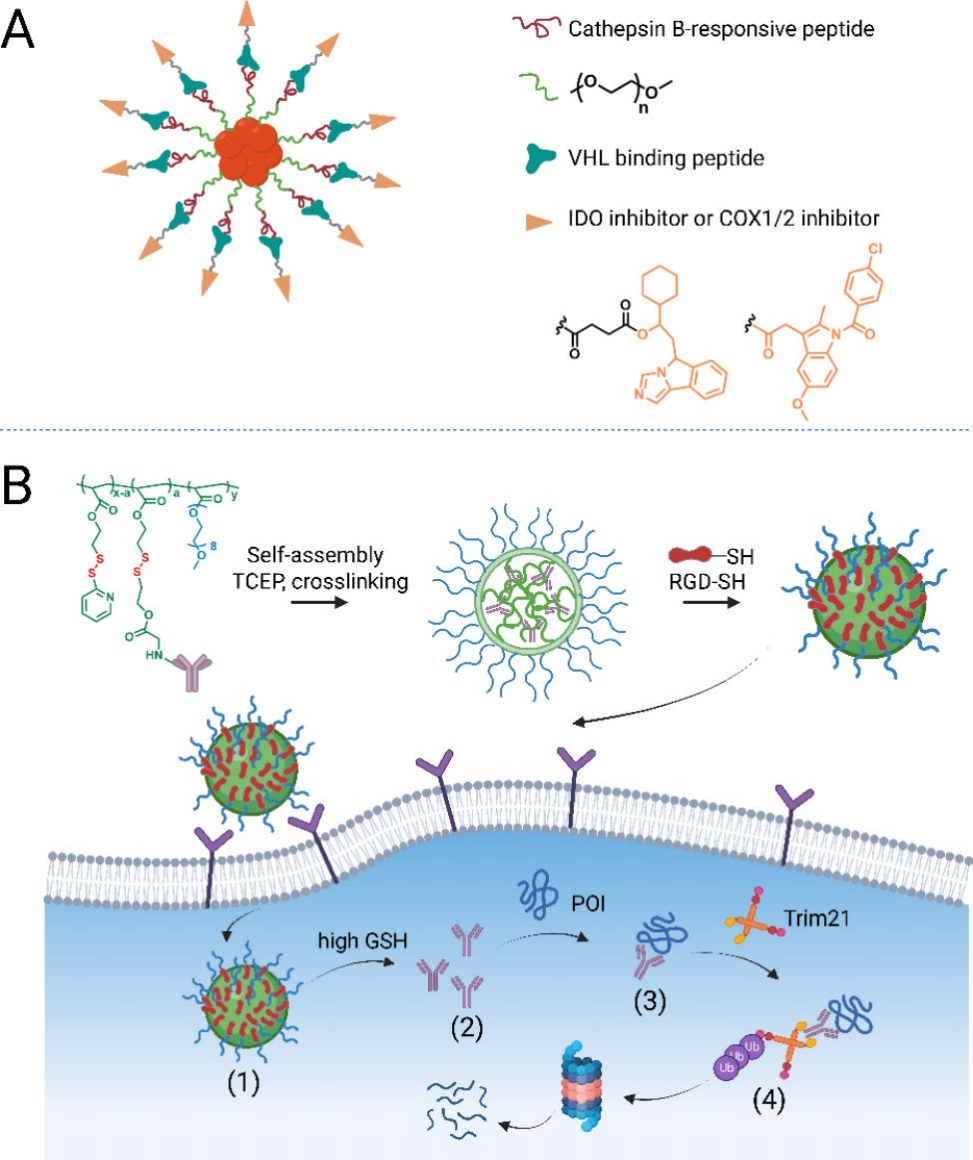
Drug delivery system to improve the delivery efficacy of bioTPD. (**A**) A polymeric nanoparticle-mediated delivery system was used for combination therapy of phototherapy, and PROTAC-engaged immunotherapy. (**B**) The antibody-tethered Nano-ERASER was transported into cells (1) and subsequently released antibodies upon a high level of glutathione (GSH) (2). The antibodies primed the corresponding POI (3) and drive them for degradation through Trim-Away pathway (4). The figure was created in BioRender.com

Most peptide-based degraders have poor cell permeability, low stability, and unfavorable pharmacokinetics. By taking advantage of nano-delivery, these bottlenecks can be surpassed. Zhang et al. [224] reported a nano-PROTACs system that conjugated a small-molecule-peptide PROTAC to a synthetic semiconducting polymeric nanoparticle via a cathepsin B-susceptible fragment (Fig. 5A). The indoleamine 2,3-dioxygenase (IDO)-targeting PROTAC is composed of a VHL-binding peptide and an IDO inhibitor (NLG919). The termed nano-PROTACs integrated phototherapeutic effects derived from the semiconducting polymers and controllable protein degradation for photo-immunometabolic anti-cancer therapy. Benefiting from the rational design of the polymer construct, the nano-PROTACs exhibited a prolonged circulation time and accumulated in the tumor site as a result of the enhanced permeability and retention (EPR) effect of the nano-sized particles. In addition, the nanoparticle exhibited excellent cellular uptake. After entry into tumor cells, the PROTAC molecule was gradually released upon recognition by cathepsin B and validly induced elimination of IDO through the proteasomal degradation pathway, eventually boosting antitumor T-cell immunity in 4T1-bearing mice. Later, they employed a similar polymeric nanosystem to deliver a cyclooxygenase 1/2 (COX1/2)-targeting PROTAC, and finally reshaped the immunosuppressive tumor microenvironment and reinforced the anti-cancer immunotherapy [225]. These studies corroborate that drug delivery systems can promote the drug-like property of peptide-based bioTPD.

As mentioned previously, Trim-Away is a rapid, highly-selective bioTPD platform [16]. Nevertheless, the Trim-Away method is severely hindered by the poor cell penetration of antibodies. To overcome this barrier, several studies used microinjection or electroporation, which is unfavorable for further clinical translation. In 2021, Sui et al. [226] developed a practical, secure version of Trim-Away termed Nano-ERASER by engrafting an antibody-conjugated polymer nanogel to deliver and release antibodies in the cytosol, which could be served as an alternative option for microinjection and electroporation. The antibody-tethered Nano-ERASER was transported into cells through receptor-mediated endocytosis and subsequently released the antibody upon a high level of glutathione (GSH) (Fig. 5B). Two POIs, GFP and coatomer protein complex ζ1 (COPZ1), were successfully eliminated through the Trim-Away pathway, paving the way for in vivo and clinical translation of antibody-based bioTPD with poor membrane permeability.

## In Vitro selection of binders for bioTPD

The design of bioTPD starts with identifying a binder with a good affinity for a desired target. Rapid and high-throughput techniques for screening extensive repertoires of high-specific binders are important tools for the development of bioTPD. The binders are commonly peptides, antibodies, and various antibody fragments. In vitro display technologies are powerful methods for the selection of peptides and antibodies from constructed libraries. Table 3 describes the most frequently used selection platforms for peptide/antibody screening.

Phage display technology was the first in vitro display technology designed to present exogenous proteins/peptides on the bacteriophage surface as fused proteins with phage coat proteins [227]. The main principle of phage display is the physical linkage between phage phenotype and genotype, which enables one to obtain foreign protein information displayed on the phage surface according to the inserted genes [228]. There are several key processes in phage display screening, and the first one is the construction of a library, which contains huge DNA clones carrying foreign genes that encode peptides or antibody fragments. Next, these genes are cloned into the phage genome and phage particles containing foreign displayed proteins are produced in E. coli. Once constructed, the phage library is applied for selection in a process referred to as biopanning. The phage library is incubated with the immobilized antigen and bound phage clones with target molecules are collected and amplified, followed by 2–4 consecutive rounds of biopanning to enrich the phage clones with high affinity to target proteins [229]. In the end, phage clones with high affinity are selected and the inserted genes encoding foreign proteins are sequenced and analyzed to obtain the sequence information of peptides or antibody fragments potentially bound to targets. Owing to its simplicity and efficacy, phage display technology has proven to be a powerful and versatile tool to identify specific binders for antigens.

Apart from phage display, cell-surface display, and cell-free display technologies have also been applied for such purposes [229, 230]. Cell-surface display technologies have been developed in yeast, bacteria, and mammalian cells. These technologies work in a similar manner to phage display: the proteins or peptides are expressed on their surface and applied for screening processes to identify the ligands of interest. The host can replicate autonomously and have several advantages over the phage system (such as production of complex mammalian proteins and proteins with post-translational modification) [231, 232]. However, the major limitations of all cell-based display technologies are the relatively small library size and the need for specialized sorting equipment for screening [233].

Additionally, a range of cell-free display technologies has also been developed and mainly include ribosomes (mRNA), and cDNA display [234]. These methods use a similar screening process with phage display following the typical four steps: binding, washing, elution, and amplification [235]. Cell-free display technologies allow the use of library size exceeding 10^13^ variants, which is several orders of magnitude larger than those of all other display technologies [234, 236].

**Table 3:** Overview of display technologies

Screening technologies	Display principle	Selection principle	Typical library size
Phage display	Protein/peptide library is displayed on the surface of phage particles	Capture/elution	10^9–10^
Cell-surface display	Protein/peptide library is displayed on the surface of a living cell as a fusion to cell surface protein	FACS selection	10^7^ eukaryotes 10^8–10^ prokaryote
Ribosome display	Protein/peptide library is displayed on the stalled ribosome/mRNA complex	Capture/elution	10^12–14^
mRNA/cDNA display	Protein/peptide library is displayed as covalently attached peptides to its encoding mRNA/cDNA	Capture/elution	10^12–14^

## Outlook

In view of the progress in the past two decades and attention from both academia and industry, it is clear that TPD is already a highly promising therapeutic modality with exciting potential. As stated by Crews et al., ‘The past is prologue’ may be the best description of the current TPD landscape. The emergence of new TPD platforms, such as LYTAC, AbTAC, and Trim-away, has built up significant hype surrounding biological TPD technologies and is poised to revolutionize classical PROTAC by modulating more undruggable targets. Myriads of conceptual TPD designs continue to be developed and progressively open up novel avenues for clinical applications. At present, the development of bioTPDs yet remains at an early exploratory stage and will require further studies to address the prevailing issues and limitations. Future efforts should be focused on identifying the underlying degradation mechanisms for newly-emerged TPD concepts and accelerating clinical translation for bioTPD.

## Data Availability

Not applicable.

## Abbreviations

α-syn: Alpha-synuclein.
AbTAC: Antibody-based PROTAC.
AdPROM: Affinity-directed protein missile.
AID: Auxin-inducible degron.
Akt: Serine/threonine-protein kinase AKT.
APC: Allophycocyanin.
AR: Androgen receptor.
ARMeD: Antibody RING-mediated destruction.
ASGPR: Asialoglycoprotein receptor.
ATTEC: Autophagy-tethering compounds.
AUTAC: Autophagy-targeting chimera.
AUTOTAC: AUTOphagy-TArgeting Chimera.
BioPROTAC: Biological PROTAC.
BIR3: baculovirus IAP repeat 3.
BRD4: Bromodomain-containing 4.
CDK5: Cyclin-dependent kinase 5.
CHAMP: Chaperone-mediated protein degradation.
CHIP: Carboxyl-terminal Hsp70 interacting protein.
CI-M6PR: Cation-independent mannose-6-phosphate receptor.
CMA: Chaperone-mediated autophagy.
CMPD: Cell membrane penetrating structural domain.
CPP: Cell penetrating peptide.
CRBN: Cereblon.
CREPT: Cell cycle-related and expression-elevated protein in tumor.
CRISPR: Clustered regularly interspaced short palindrome repeats.
CTM: CMA targeting structural domain.
DAPK1: Death-associated protein kinase 1.
DARPin: Designed ankyrin repeat proteins.
DC_50_: Half-maximal degradation concentrations.
ERG: ETS-related gene.
ERα: Estrogen receptor alpha.
ERβ: Estrogen receptor beta.
FcRn: Neonatal Fc receptor.
FKBP: FK506- and rapamycin-binding protein.
GalNAc: N-acetyl galactosamine.
HIF: Hypoxia-inducible factor.
HyT: Hydrophobic tag.
IAP: Inhibitor of apoptosis protein.
IGFIIR: Insulin-like growth factor type II receptor.
IgG: Immunoglobulin G.
ITC: Isothermal titration calorimetry.
K_D_: Dissociation constant.
KEAP1: Kelch-1ike ECH- associated protein l.
KRAS: Kirsten rat sarcoma viral oncogene homologue.
L2A: Lysosome-associated membrane protein type 2 A.
LBs: Lewy bodies.
LEF1: lymphoid enhancer-binding factor 1.
LID: Ligand-Induced Degradation.
LSS: Lysosomal sorting sequence.
LYTAC: Lysosome-targeting chimeras.
MADTAC: Macroautophagy Degradation Targeting Chimeras.
MDM2: Mouse double minute-2.
Met: Mesenchymal epithelial transition.
MetAP2: Methionine aminopeptidase 2.
MIF: Macrophage migration inhibitory factor.
MOG: Myelin oligodendrocyte glycoprotein.
NF-κB: Nuclear factor kappa-light-chain enhancer of activated B cells.
NR2B: N-methyl-D-aspartate receptor 2B.
Nrf2: Transcription factor NF-E2-related factor 2.
PBD: Target protein binding structural domain.
PCC: Protein-catalyzed capture.
PCNA: Proliferating cell nuclear antigen.
PD-1: Programmed cell death 1.
PDAC: Pancreatic ductal adenocarcinoma.
PD-L1: Programmed cell death ligand 1.
PI3K: Phosphatidylinositol-3-kinase.
PIPK2: Receptor Interacting Serine/Threonine Kinase 2.
PK: Pharmacokinetics.
POI: Protein of interest.
PROTAC: PROteolysis-TArgeting Chimeras.
PTK-7: Protein kinase-like 7.
RIBOTAC: Ribonuclease targeting chimera.
RING: Really Interesting New Gene.
RNF4: Ring Finger Protein 4.
SCF: SKP1, CUL1, and F-box protein.
SHP2: SH2-containing protein tyrosine phos-phatase 2.
SMPI: Small molecule protein hydrolysis inducer.
SNIPERs: Specific and Non-genetic IAP-dependent Protein Erasers.
SPOP: Speckle-type BTB/POZ protein.
SUMO: Small ubiquitin-like modifier.
Tau: microtubule-associated protein.
TBK1: TANK-binding kinase 1.
TPD: Targeted protein degradation.
TRAFTAC: TRAnscription Factor Targeting Chimeras.
TRIM21: Tripartite motif-containing protein 21.
TRIM24: Tripartite motif containing 24.
VHL: Von Hippel-Lindau.
